# Supplementary material for: Pan-cancer ion transport signature reveals functional regulators of glioblastoma aggression
Source: EMBO J. 2024 Jan 2;43(2):196–224. doi: 10.1038/s44318-023-00016-x (PMC10897389; doi:10.1038/s44318-023-00016-x)
Supplement: Supplementary file 1 — Appendix [file 44318_2023_16_MOESM1_ESM.pdf]

APPENDIX: Pan-cancer ion transport signature reveals functional regulators of glioblastoma aggression

Alexander T. Bahcheli [*], Hyun-Kee Min [*], Masroor Bayati, Hongyu Zhao, Alexander Fortuna, Weifan Dong, Irakli Dzneladze, Jade Chan, Xin Chen, Kissy Guevara-Hoyer, Peter B. Dirks, Xi Huang [@], Jüri Reimand [@]

[*] These authors contributed equally to this work

[@] correspondence: Juri.Reimand@utoronto.ca, Xi.Huang@Sickkids.ca

EMBO Journal (2023)

Table of Contents.

Appendix Figure	Page
**Appendix Figure S1.** Significantly elevated expression of IP genes in subsets of cancer samples.	3
**Appendix Figure S2.** Machine learning identifies IP genes associated with OS in GBM.	4
**Appendix Figure S3.** Prioritised IP gene expression associates with patient survival.	5
**Appendix Figure S4.** *GJB2* and *SCN9A* expression is associated with patient OS in GBM.	6
**Appendix Figure S5.** Differential gene expression analysis of samples with high *vs*. low expression of *GJB2* and *SCN9A* in TCGA.	7
**Appendix Figure S6.** *GJB2* knockdown in GBM cells shortens TNT length, filopodia length, and lifetime in live cells.	8
**Appendix Figure S7.** Subcellular localizations of *GJB2* and *SCN9A*.	9

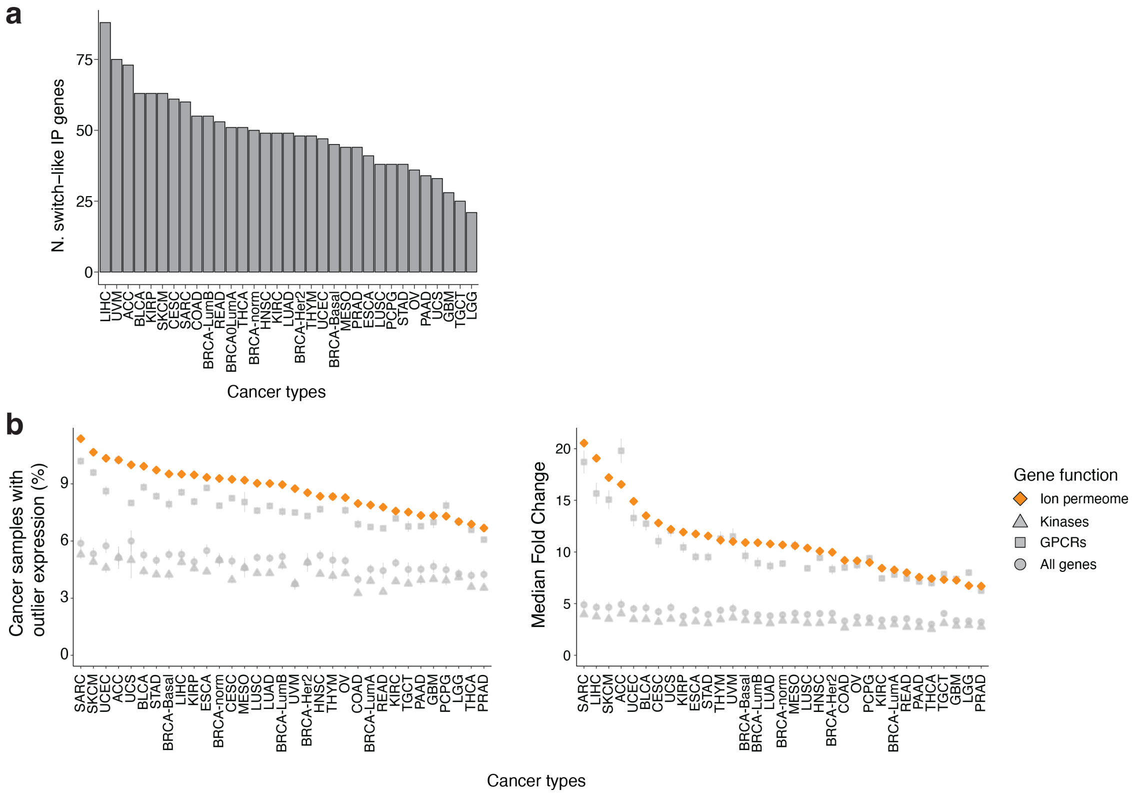

Appendix Figure S1. Significantly elevated expression of IP genes in subsets of cancer samples. (a) Numbers of IP genes with switch-like expression patterns in cancer types. Bar plot shows the number of genes for which the median expression in given cancer type was zero while a minority of samples showed elevated IP gene expression. **(b)** Analysis of elevated IP gene expression in cancer types in TCGA using permutation tests. IP genes (orange) show a higher fractions of cancer samples with highly elevated expression (left) and higher increase in expression relative to other samples of the same cancer type (right). Control genes (gray) include all protein-coding genes (circles) and two major classes of drug targets: kinases and GPCRs (triangles and squares, respectively). Control gene sets (276 genes) were sampled randomly for 10,000 iterations to match the number of IP genes we analysed and median values are shown. Error bars show one standard deviation.

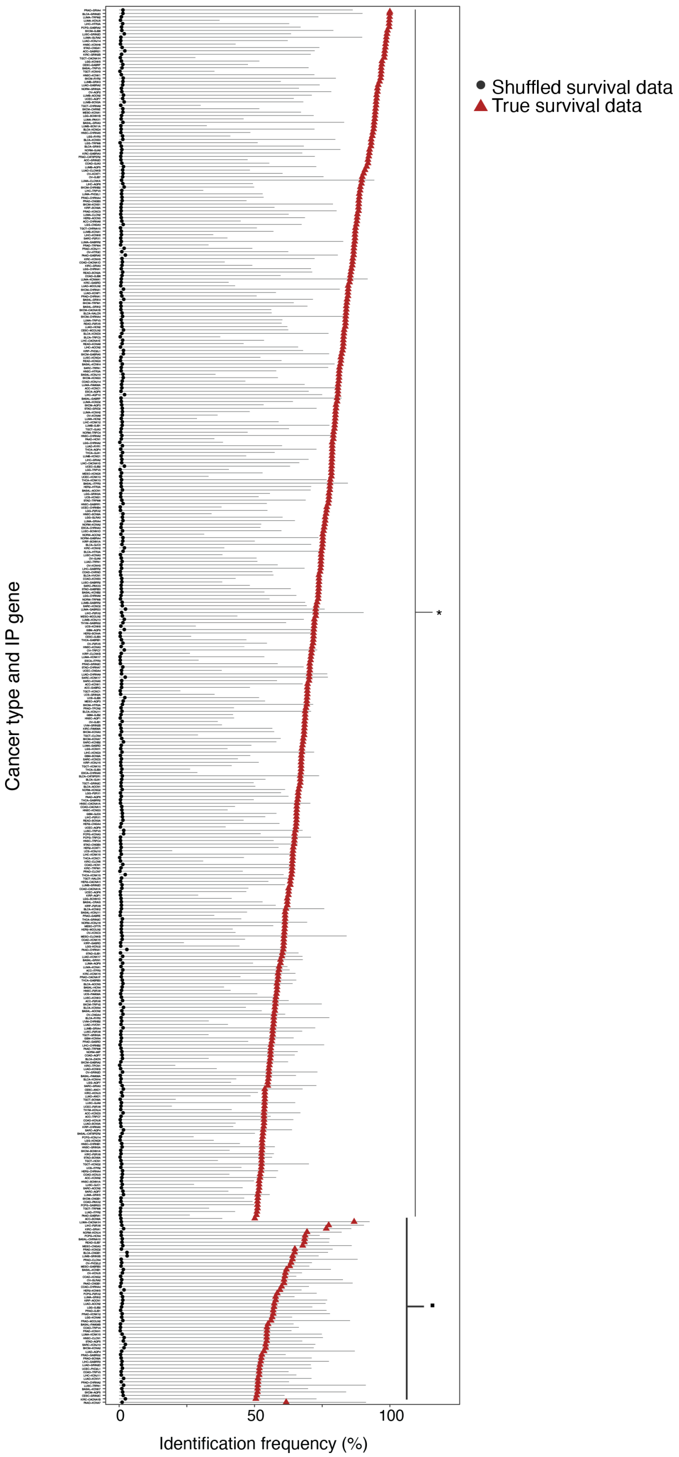

Appendix Figure S2. Machine learning identifies IP genes associated with OS in GBM. Selection of survival-associated IP genes identifies genes associated with patient survival significantly more frequently than expected from permuted survival datasets. The feature selection pipeline was run repeatedly to discover survival-associated IP genes on randomly shuffled patient survival data. Survival data was randomly permuted among patients over a series of 100 iterations. The frequencies of IP genes identified in true data were compared to 100 iterations of shuffled data. Empirical *P*-values are shown (• $P < 0.1$, * $P < 0.05$).

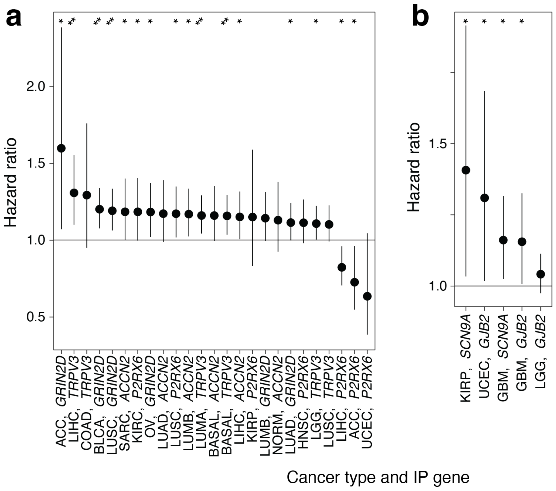

Appendix Figure S3. Prioritised IP gene expression associates with patient survival. Multivariate HR values of IP genes prioritised in several cancer types (a) and in GBM (b). CoxPH survival models were trained on single IP gene expression and relevant clinical variables (age, sex, tumor stage and grade, and *IDH1/2* mutation status). Median multivariate HR is shown with 95% confidence intervals. Wald P-values are shown (*$P < 0.05$, **$P < 0.01$).

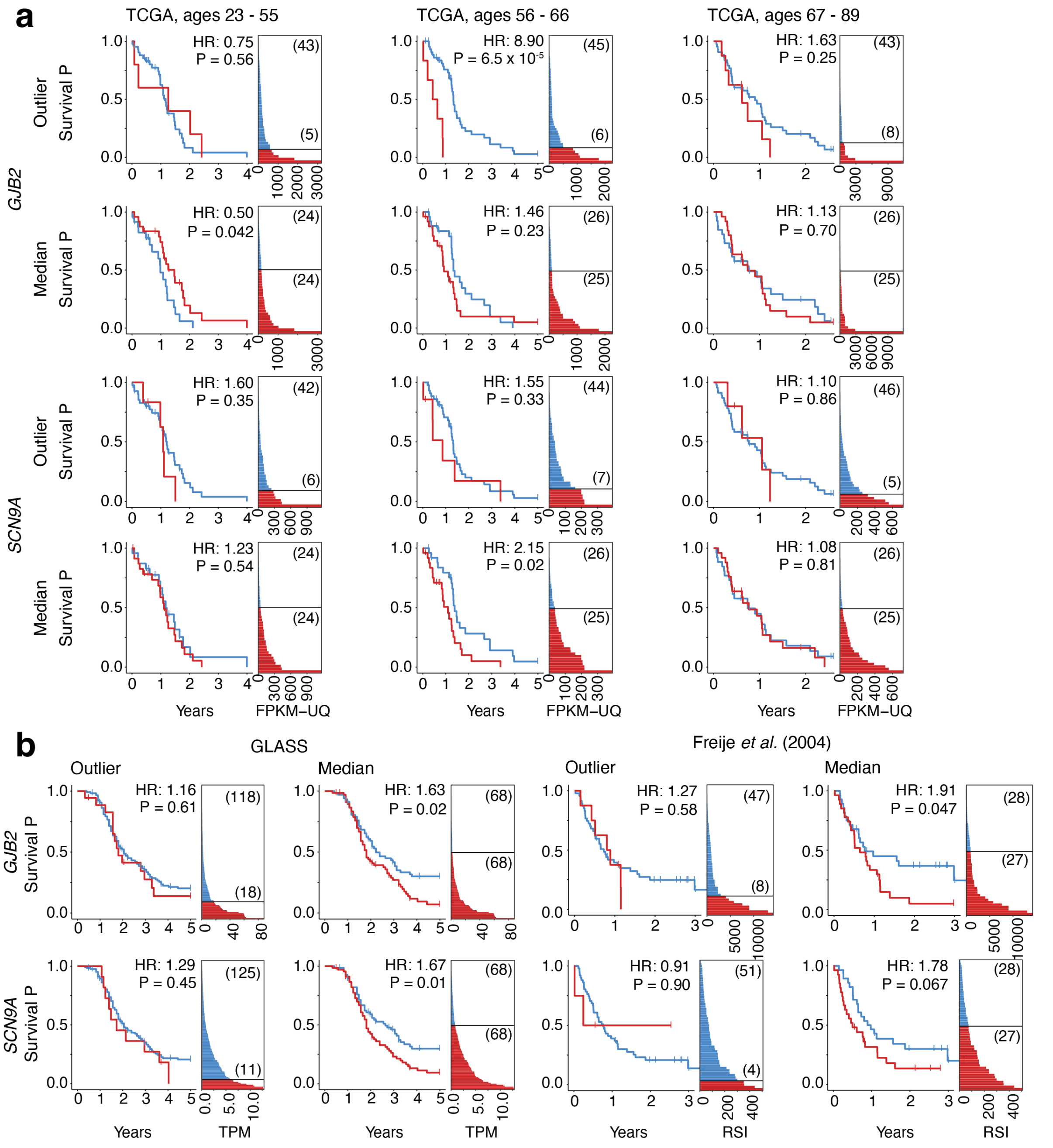

Appendix Figure S4. *GJB2* and *SCN9A* expression is associated with patient OS in GBM. Kaplan-Meier curves for overall survival (OS) in TCGA **(a)** and two independent cohorts **(b)**. Samples were split into high or low risk groups by their expression levels by either median-based or outlier cut-off. For each plot, Kaplan-Meier survival curves (left) and sample expression bar plots (right) are shown, along with sample counts in groups and HR and P-values (P) for univariate CoxPH survival models trained on risk group classifications. For the TCGA cohort, patients were split by age into three approximately equal groups.

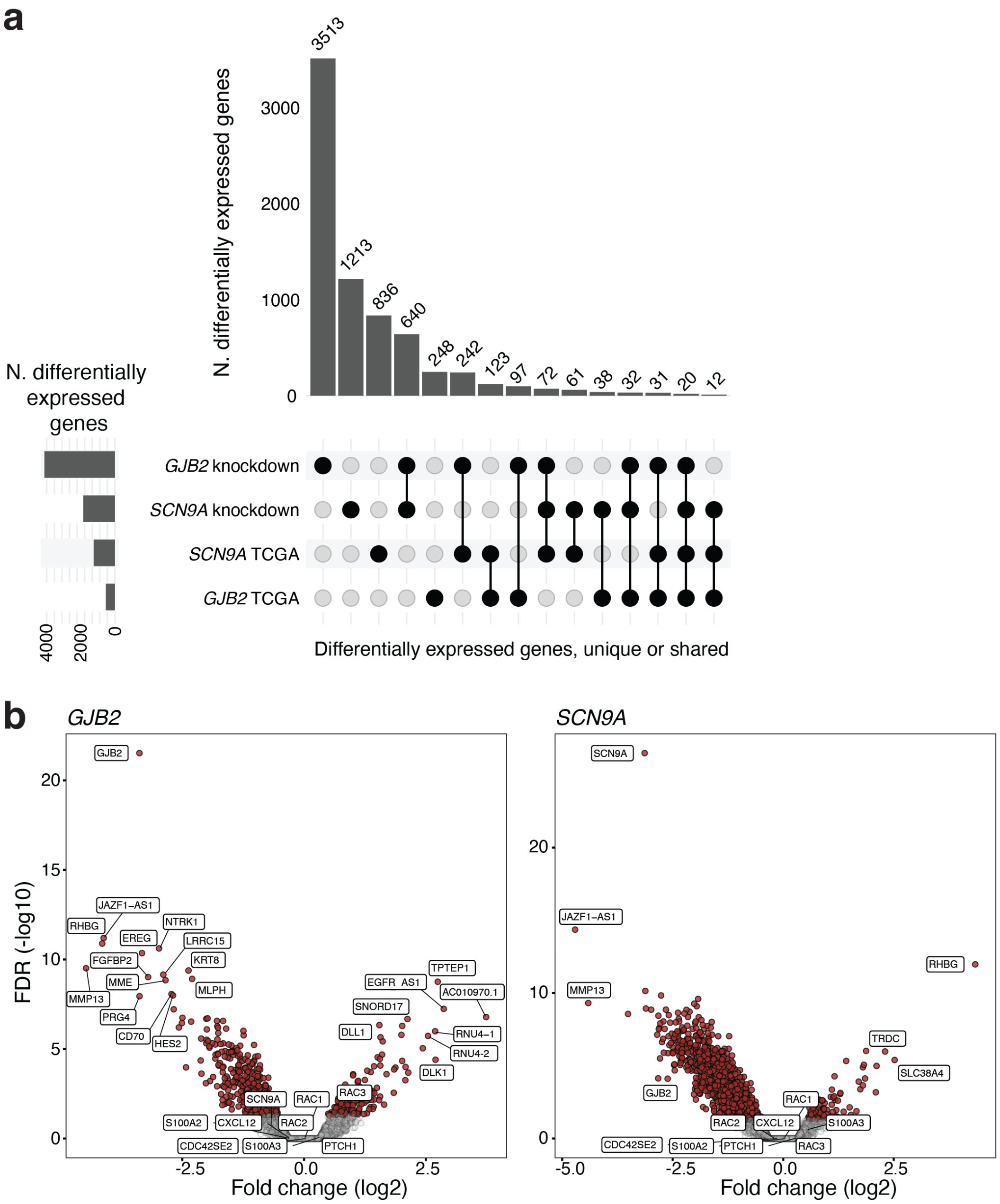

Appendix Figure S5. Differential gene expression analysis of GBMs with high *vs*. low expression of *GJB2* and *SCN9A* in TCGA and comparison with knockdown experiments. (a) Upset plot compares differentially expressed genes in patient GBMs in TCGA **(**panel b**)** with genes identified in *GJB2* or *SCN9A* knockdown experiments in patient-derived GBM cell lines. Bars show the number of genes found in knockdown experiments or in patient GBMs. Dot-and-lines plot below shows differentially expressed genes shared or unique in the four conditions. Horizontal bars show gene counts. **(b)** Volcano plot of differentially expressed genes found in patient GBMs in TCGA grouped by median expression of *GJB2* (left) or *SCN9A* (right). Significant genes are shown in red (EdgeR; FC > 1.25, FDR < 0.05) and top genes or genes associated with tunnelling nanotubes are labelled.

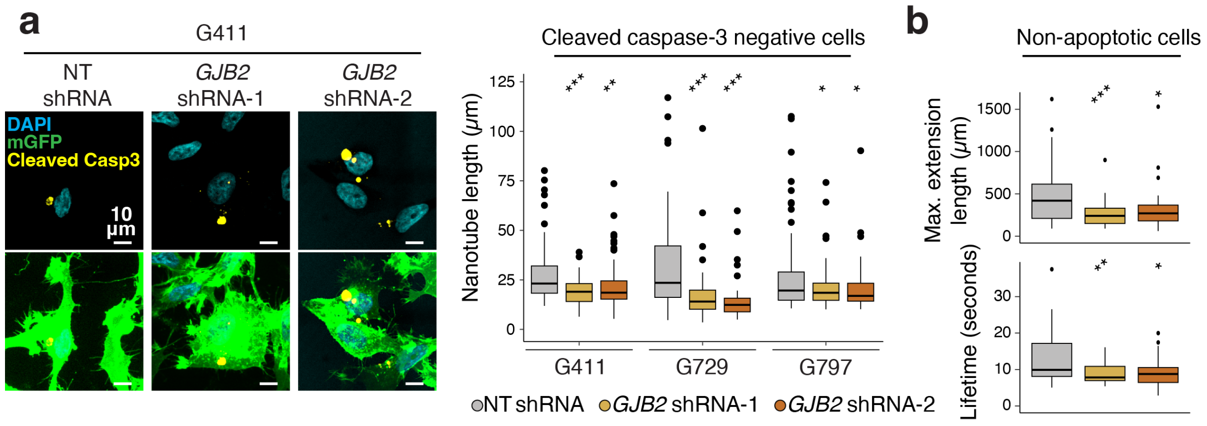

Appendix Figure S6. ***GJB2* knockdown in GBM cells shortens TNT length, filopodia length, and lifetime in live cells**. **(a)** TNT projection length is reduced in live *GJB2* knockdown cells. Apoptotic cells were identified by immunofluorescence imaging of the apoptosis marker cleaved caspase-3 (left). TNT length was quantified from confocal microscopy images of *GJB2* knockdown cells labeled with mGFP, and negative for cleaved caspase-3 (right). **(b)** Filopodia length and lifetime are reduced in live *GJB2* knockdown cells. mGFP-expressing GBM cells were live-imaged for 2.5 hours. Maximum filopodia length and lifetime were measured throughout the imaging period. Cells exhibiting apoptotic rounding or blebbing were excluded from quantification. All results represent three independent replicates. *P* values of U tests are shown (* < 0.05, ** < 0.01, *** < 0.001). Box plots span the interquartile range (IQR; 25th-75th percentiles) where median values are shown as lines and whiskers reflect values within 1.5x of IQR.

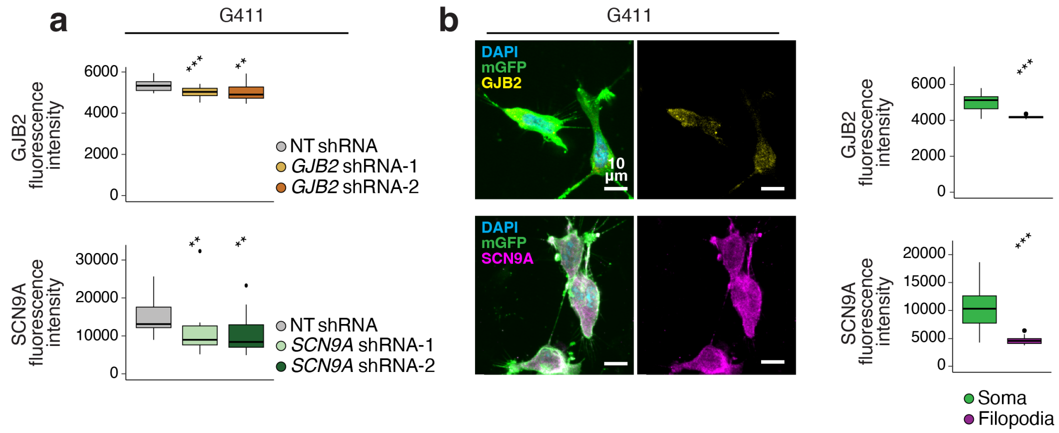

Appendix Figure S7. Subcellular localizations of GJB2 and SCN9A. (a) GJB2 and SCN9A antibodies confirm reduced protein expression in knockdown experiments. Immunofluorescence imaging of GJB2 and SCN9A was performed in mGFP G411 cells 4 days post lentiviral shRNA transduction. Fluorescence intensities were quantified in NT control or knockdown cells. **(b)** GJB2 and SCN9A exhibit broad membrane localization in the soma and low presence in filopodia. All results represent three independent replicates. *P* values of paired Welch's t-tests are shown (* < 0.05, ** < 0.01, *** < 0.001). Box plots span the interquartile range (IQR; 25th-75th percentiles) where median values are shown as lines and whiskers reflect values within 1.5x of IQR
