# Supplementary figures and images for: Pan-cancer ion transport signature reveals functional regulators of glioblastoma aggression
Source: EMBO J. 2024 Jan 2;43(2):196–224. doi: 10.1038/s44318-023-00016-x (PMC10897389; doi:10.1038/s44318-023-00016-x)

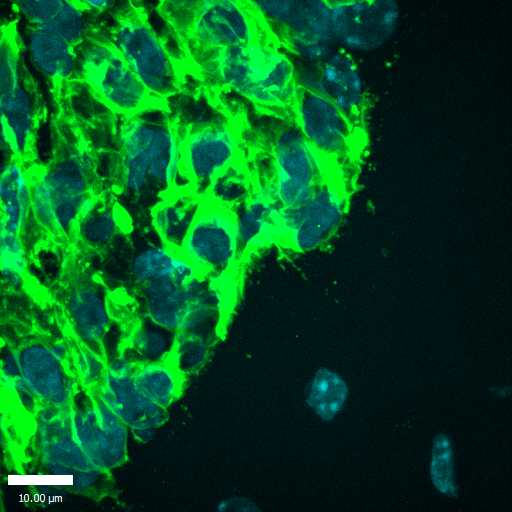

Supplement: Supplementary file 13 — Source Data Fig. 5 [file 44318_2023_16_MOESM13_ESM.zip › Figure 5/5G/GJB2 shRNA #1.tif]

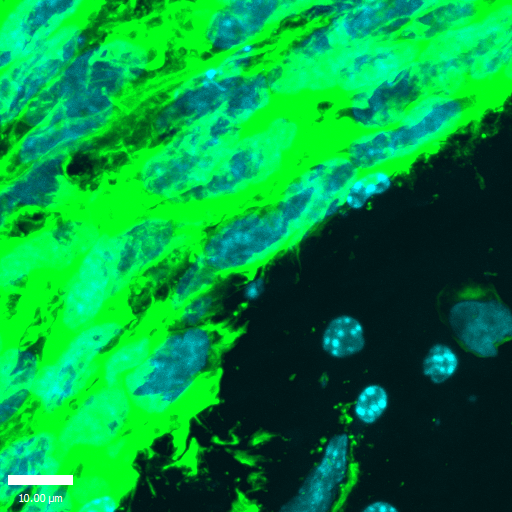

Supplement: Supplementary file 13 — Source Data Fig. 5 [file 44318_2023_16_MOESM13_ESM.zip › Figure 5/5G/GJB2 shRNA #2.tif]

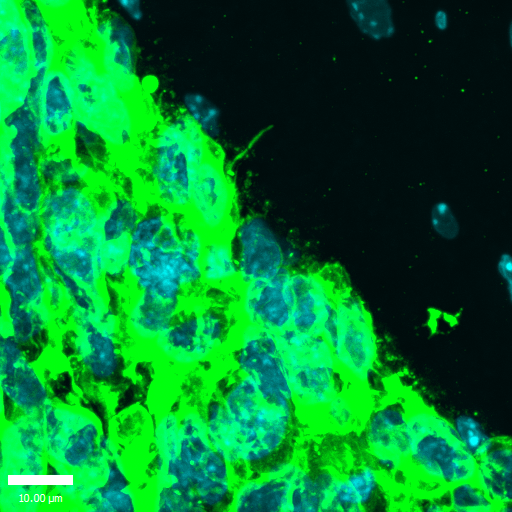

Supplement: Supplementary file 13 — Source Data Fig. 5 [file 44318_2023_16_MOESM13_ESM.zip › Figure 5/5G/NT shRNA.tif]

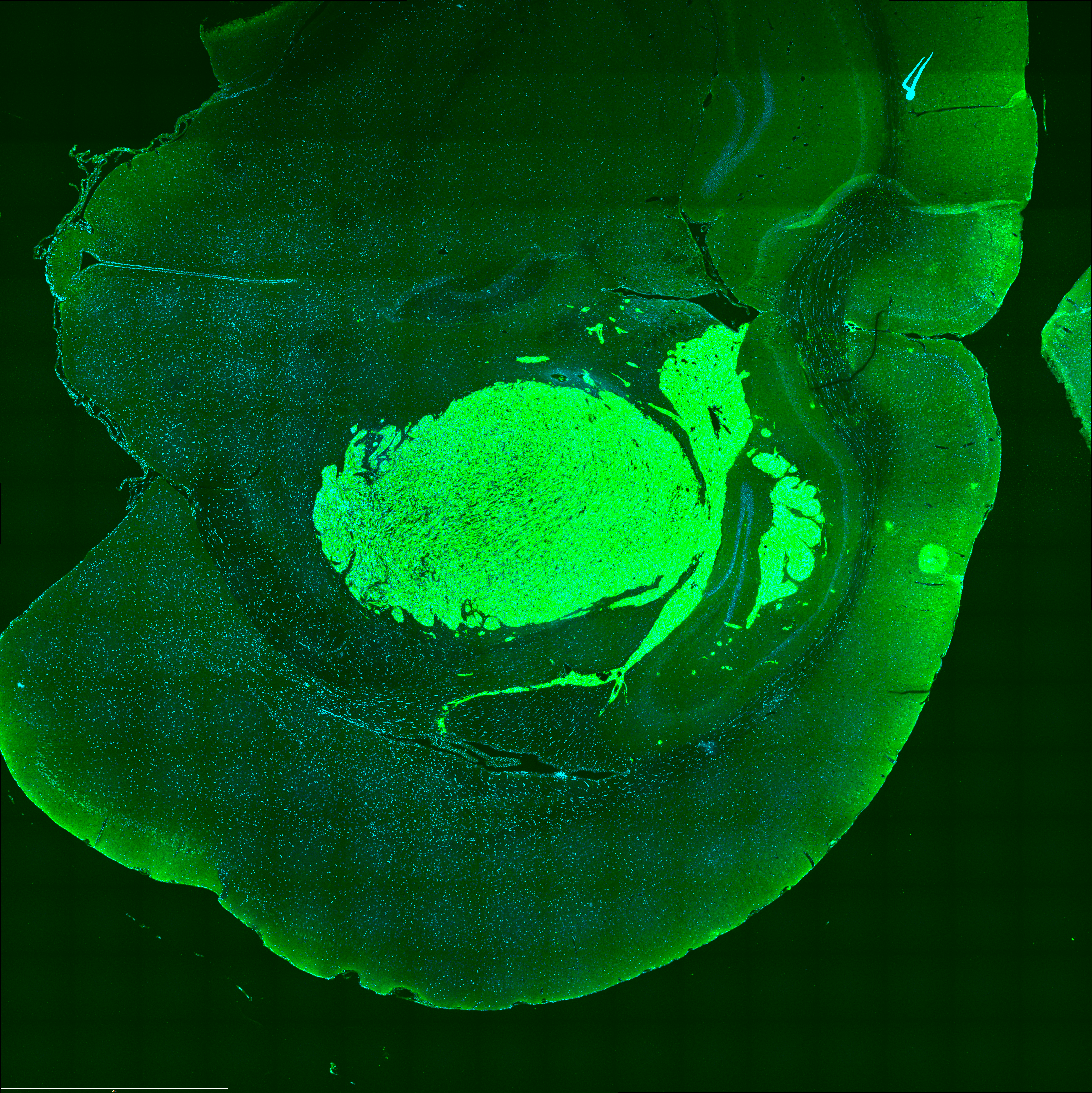

Supplement: Supplementary file 13 — Source Data Fig. 5 [file 44318_2023_16_MOESM13_ESM.zip › Figure 5/5F/NT shRNA_2.tiff]

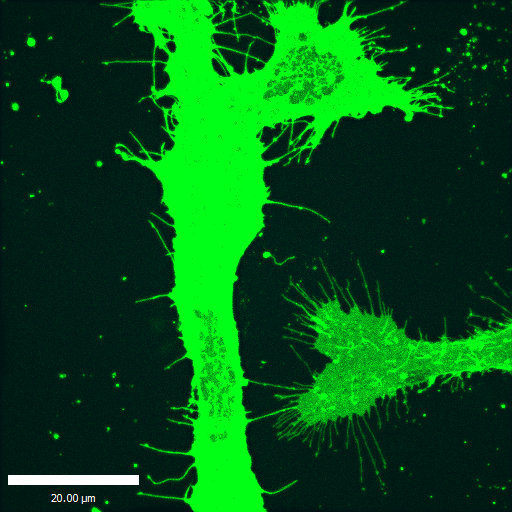

Supplement: Supplementary file 13 — Source Data Fig. 5 [file 44318_2023_16_MOESM13_ESM.zip › Figure 5/5C/G797 GJB2 shRNA #1.tif]

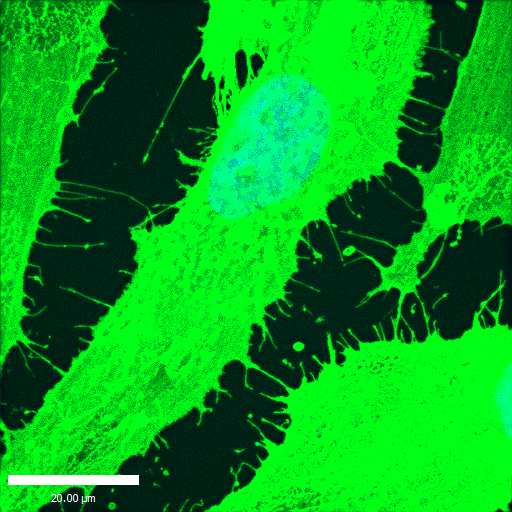

Supplement: Supplementary file 13 — Source Data Fig. 5 [file 44318_2023_16_MOESM13_ESM.zip › Figure 5/5C/G797 GJB2 shRNA #2.tif]

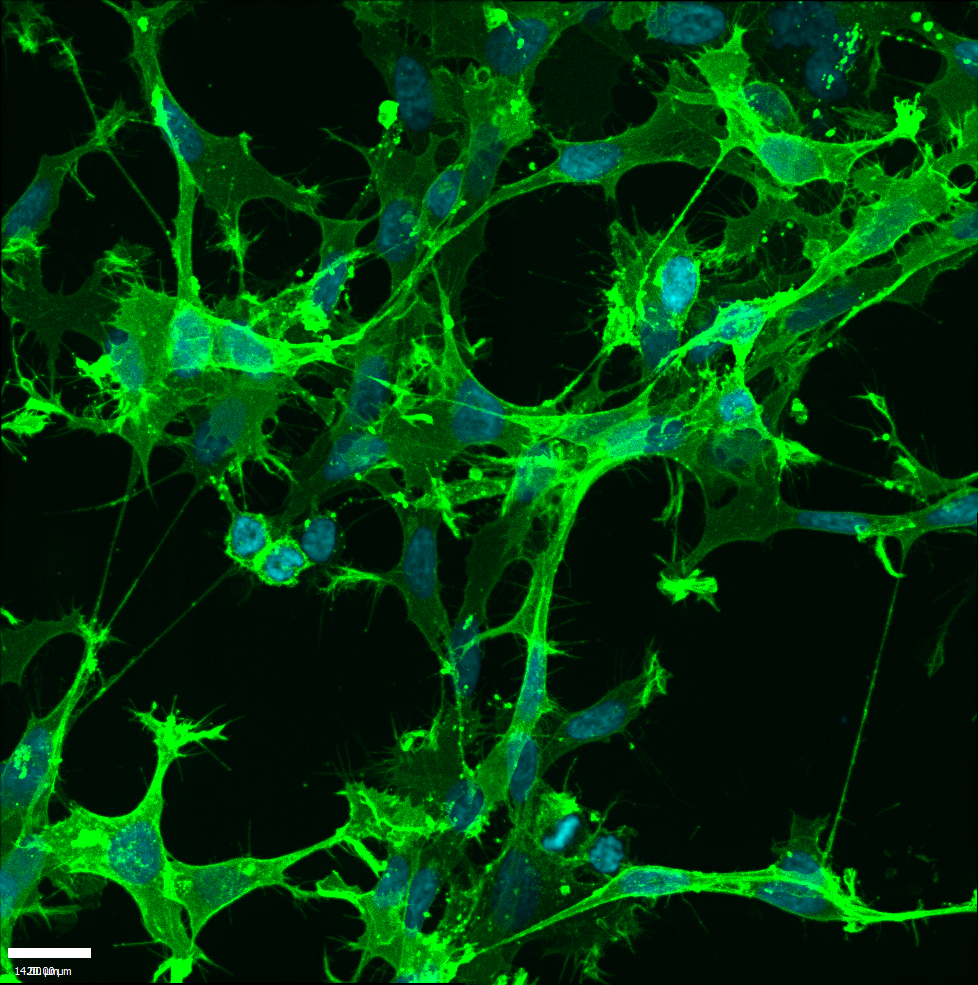

Supplement: Supplementary file 13 — Source Data Fig. 5 [file 44318_2023_16_MOESM13_ESM.zip › Figure 5/5C/G411 NT shRNA.tif]

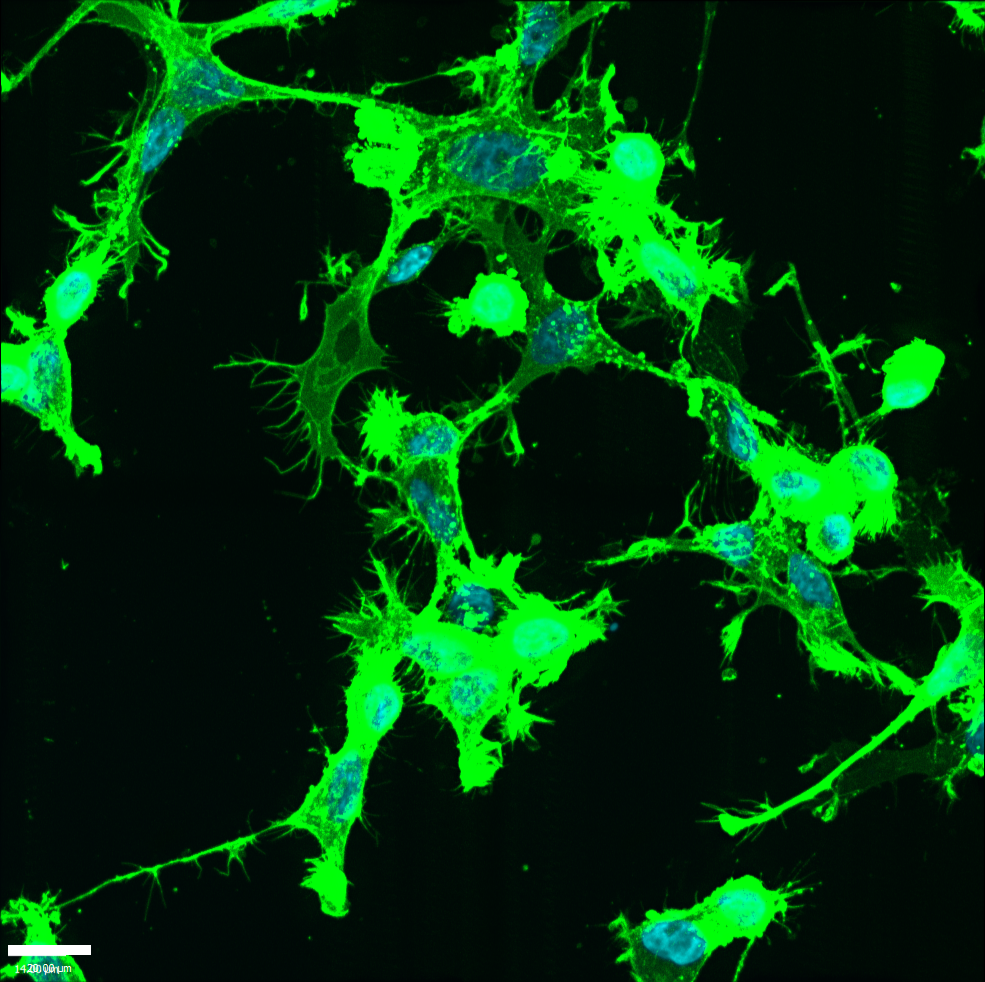

Supplement: Supplementary file 13 — Source Data Fig. 5 [file 44318_2023_16_MOESM13_ESM.zip › Figure 5/5C/G411 GJB2 shRNA #1.tif]

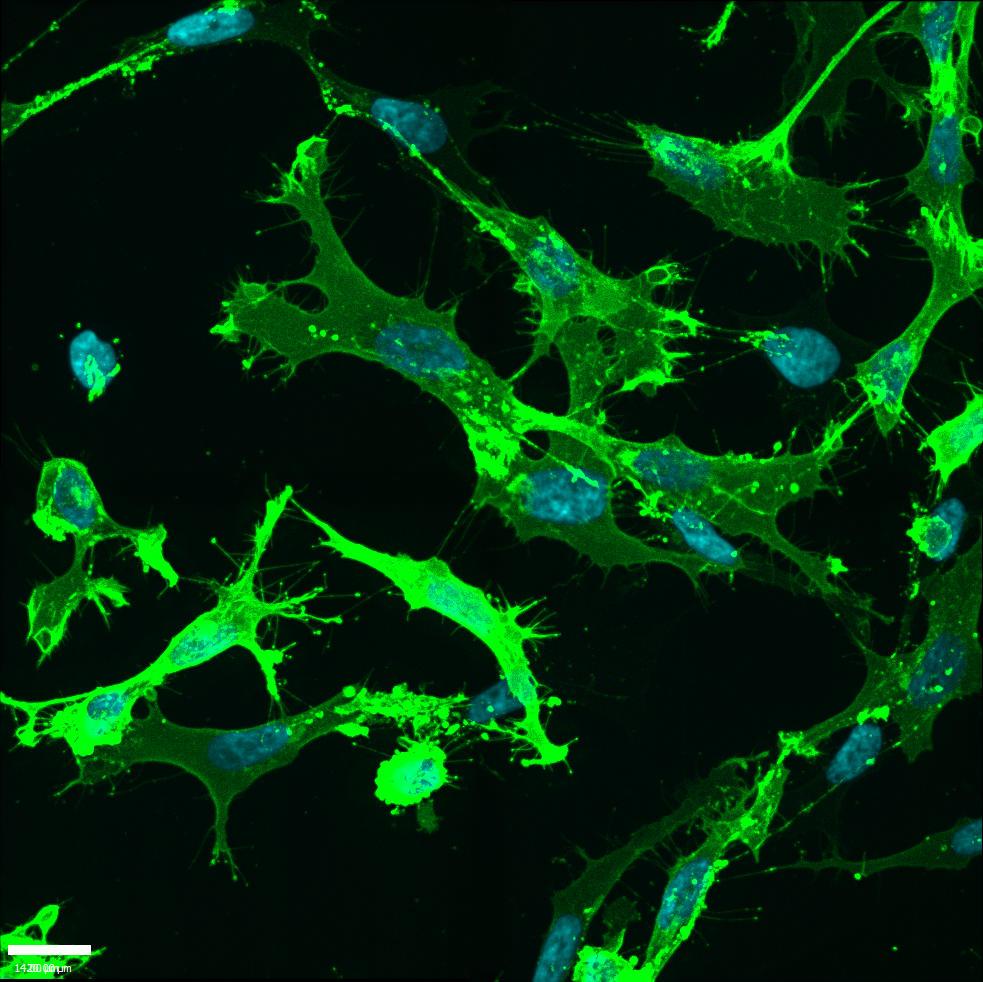

Supplement: Supplementary file 13 — Source Data Fig. 5 [file 44318_2023_16_MOESM13_ESM.zip › Figure 5/5C/G411 GJB2 shRNA #2.tif]

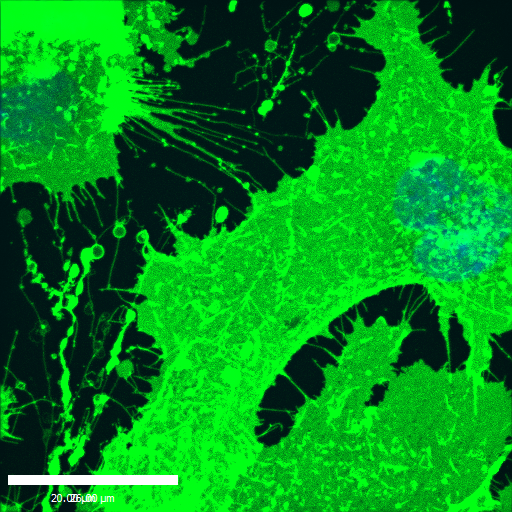

Supplement: Supplementary file 13 — Source Data Fig. 5 [file 44318_2023_16_MOESM13_ESM.zip › Figure 5/5C/G729 GJB2 shRNA #2.tif]

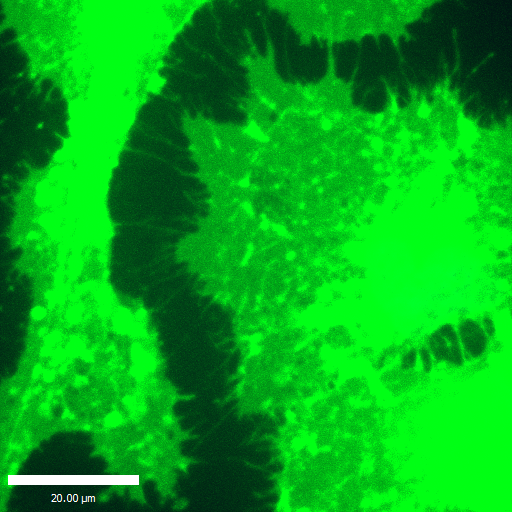

Supplement: Supplementary file 13 — Source Data Fig. 5 [file 44318_2023_16_MOESM13_ESM.zip › Figure 5/5C/G729 GJB2 shRNA #1.tif]

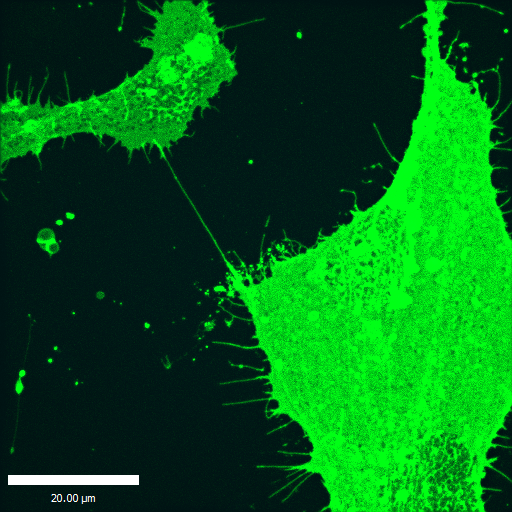

Supplement: Supplementary file 13 — Source Data Fig. 5 [file 44318_2023_16_MOESM13_ESM.zip › Figure 5/5C/G797 NT shRNA.tif]

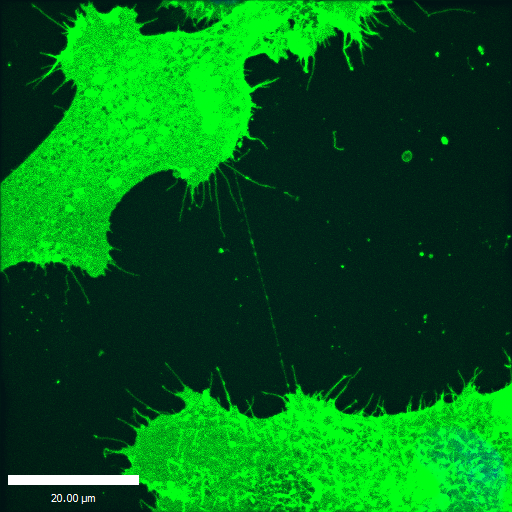

Supplement: Supplementary file 13 — Source Data Fig. 5 [file 44318_2023_16_MOESM13_ESM.zip › Figure 5/5C/G729 NT shRNA.tif]

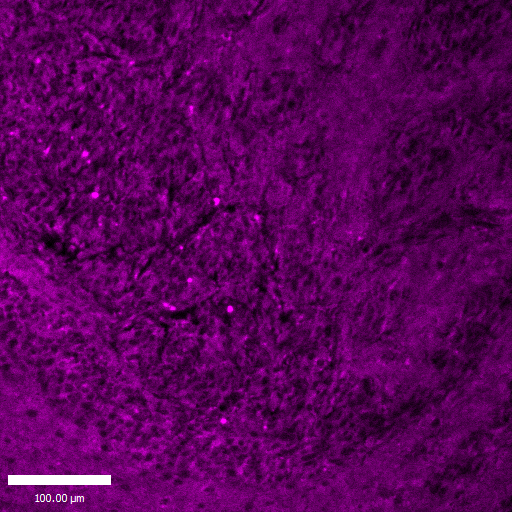

Supplement: Supplementary file 13 — Source Data Fig. 5 [file 44318_2023_16_MOESM13_ESM.zip › Figure 5/5E/NT shRNA Rac1.tif]

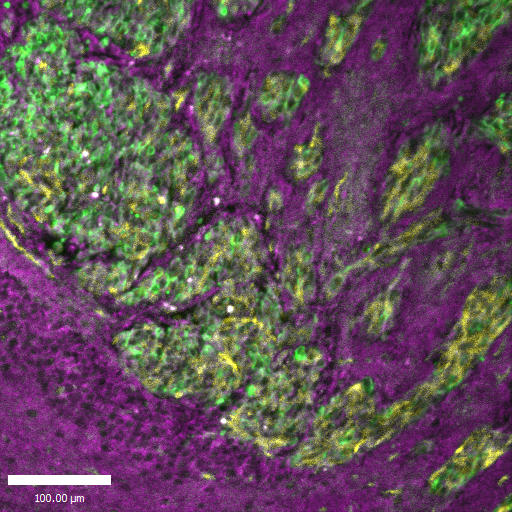

Supplement: Supplementary file 13 — Source Data Fig. 5 [file 44318_2023_16_MOESM13_ESM.zip › Figure 5/5E/NT shRNA merge.tif]

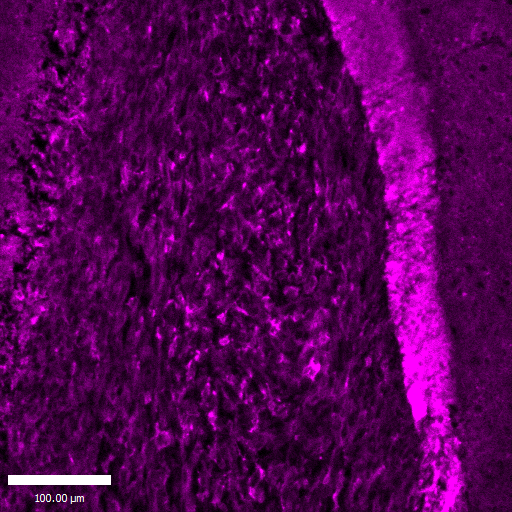

Supplement: Supplementary file 13 — Source Data Fig. 5 [file 44318_2023_16_MOESM13_ESM.zip › Figure 5/5E/GJB2 shRNA #2 RAC1.tif]

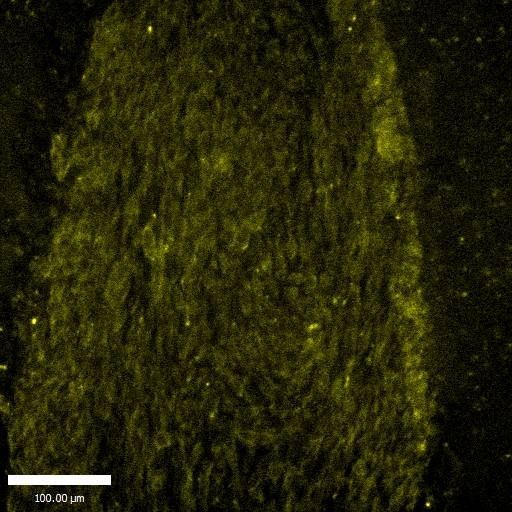

Supplement: Supplementary file 13 — Source Data Fig. 5 [file 44318_2023_16_MOESM13_ESM.zip › Figure 5/5E/GJB2 shRNA #2 pMLC2.tif]

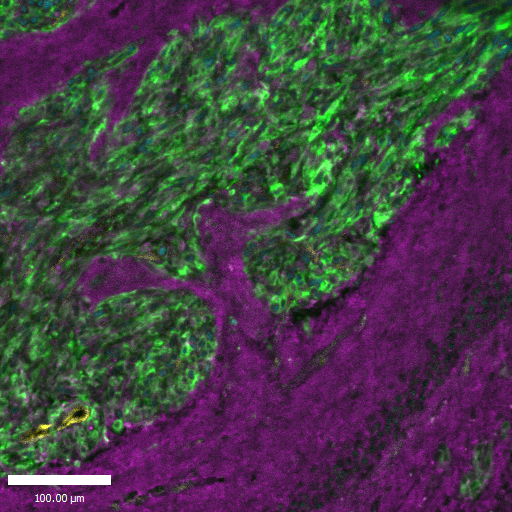

Supplement: Supplementary file 13 — Source Data Fig. 5 [file 44318_2023_16_MOESM13_ESM.zip › Figure 5/5E/GJB2 shRNA #1 merge.tif]

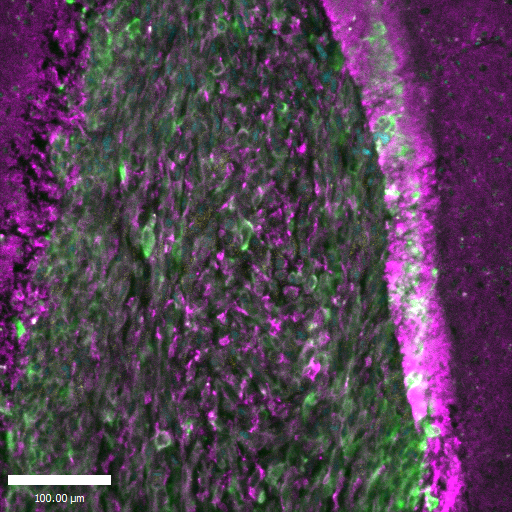

Supplement: Supplementary file 13 — Source Data Fig. 5 [file 44318_2023_16_MOESM13_ESM.zip › Figure 5/5E/GJB2 shRNA #2 merge.tif]

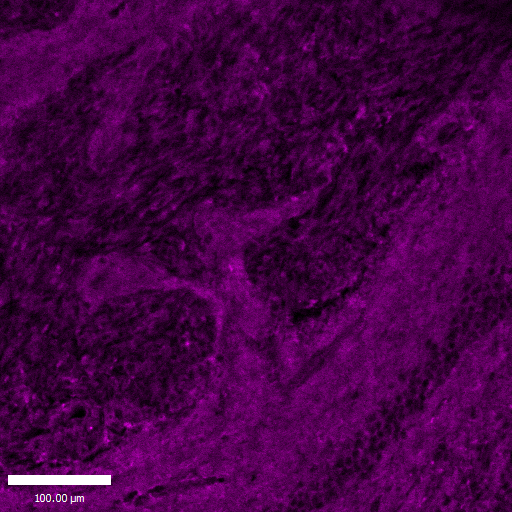

Supplement: Supplementary file 13 — Source Data Fig. 5 [file 44318_2023_16_MOESM13_ESM.zip › Figure 5/5E/GJB2 shRNA #1 RAC1.tif]

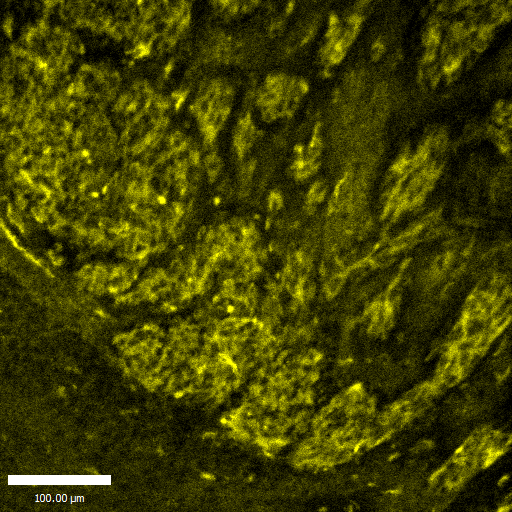

Supplement: Supplementary file 13 — Source Data Fig. 5 [file 44318_2023_16_MOESM13_ESM.zip › Figure 5/5E/NT shRNA pMLC2.tif]

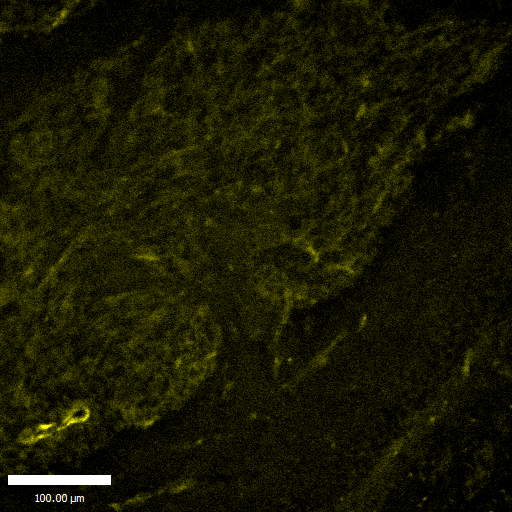

Supplement: Supplementary file 13 — Source Data Fig. 5 [file 44318_2023_16_MOESM13_ESM.zip › Figure 5/5E/GJB2 shRNA #1 pMLC2.tif]

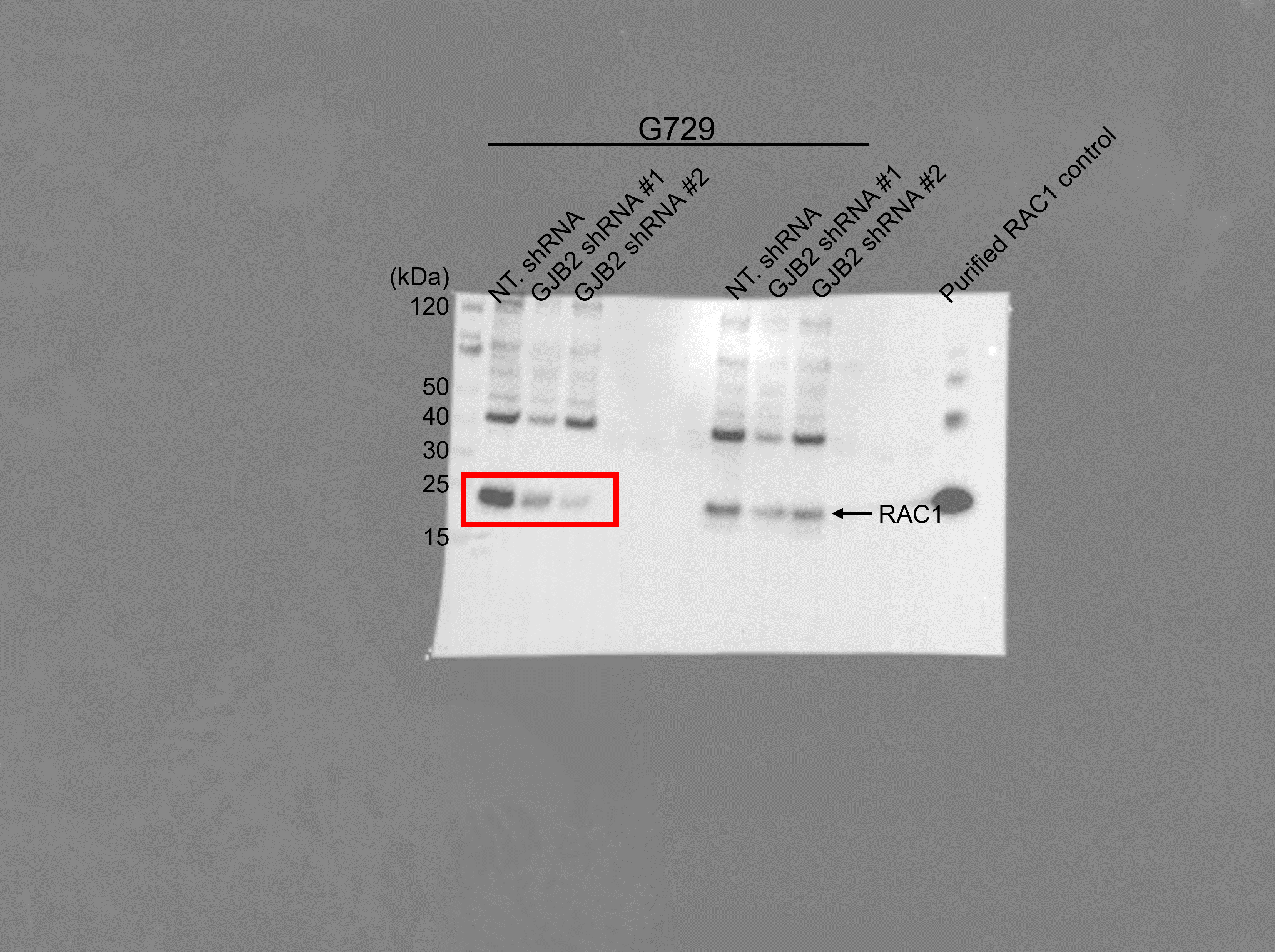

Supplement: Supplementary file 13 — Source Data Fig. 5 [file 44318_2023_16_MOESM13_ESM.zip › Figure 5/5B/G729_2 replicates.png]

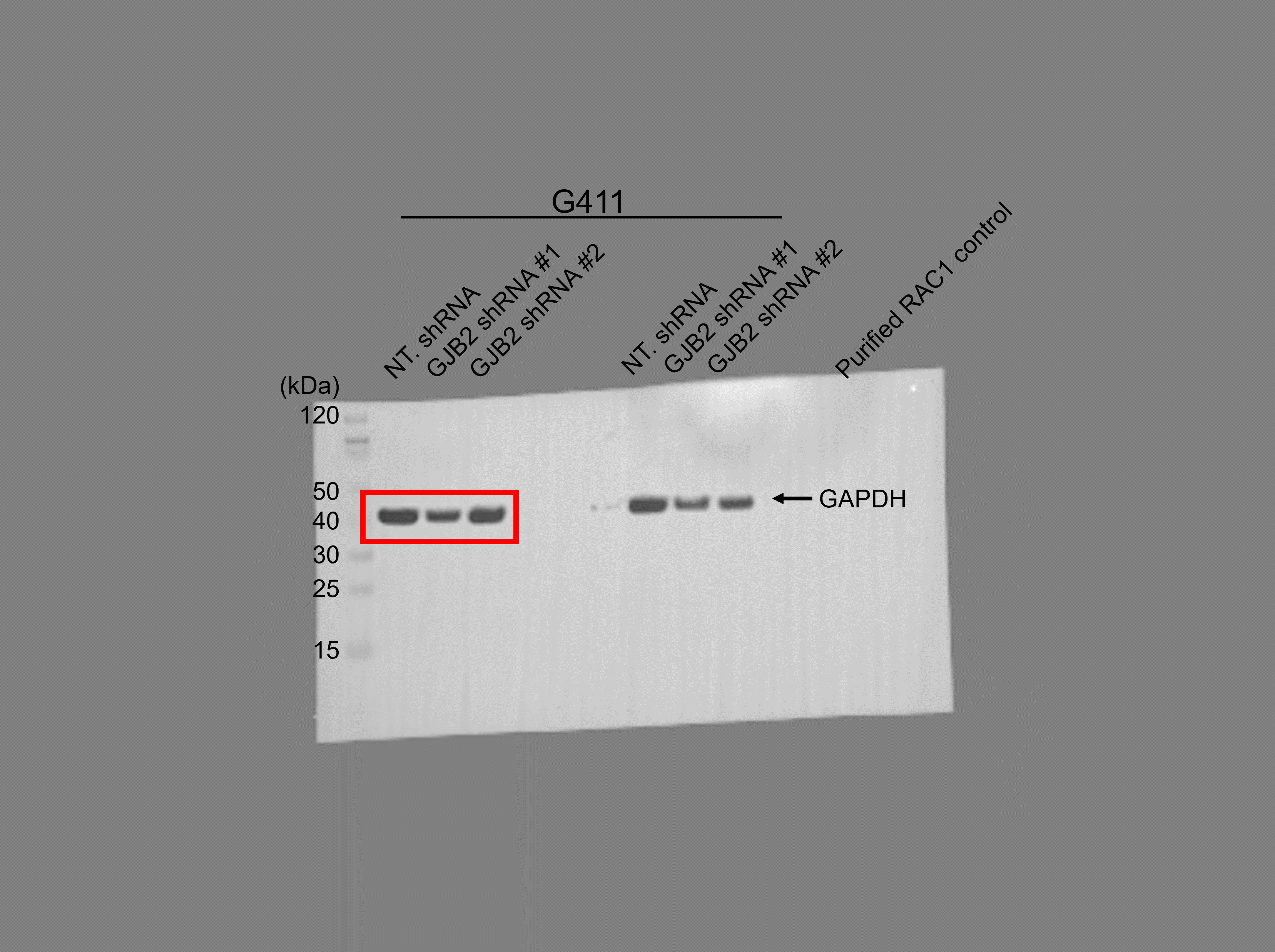

Supplement: Supplementary file 13 — Source Data Fig. 5 [file 44318_2023_16_MOESM13_ESM.zip › Figure 5/5B/G411_2 replicates_GAPDH.png]

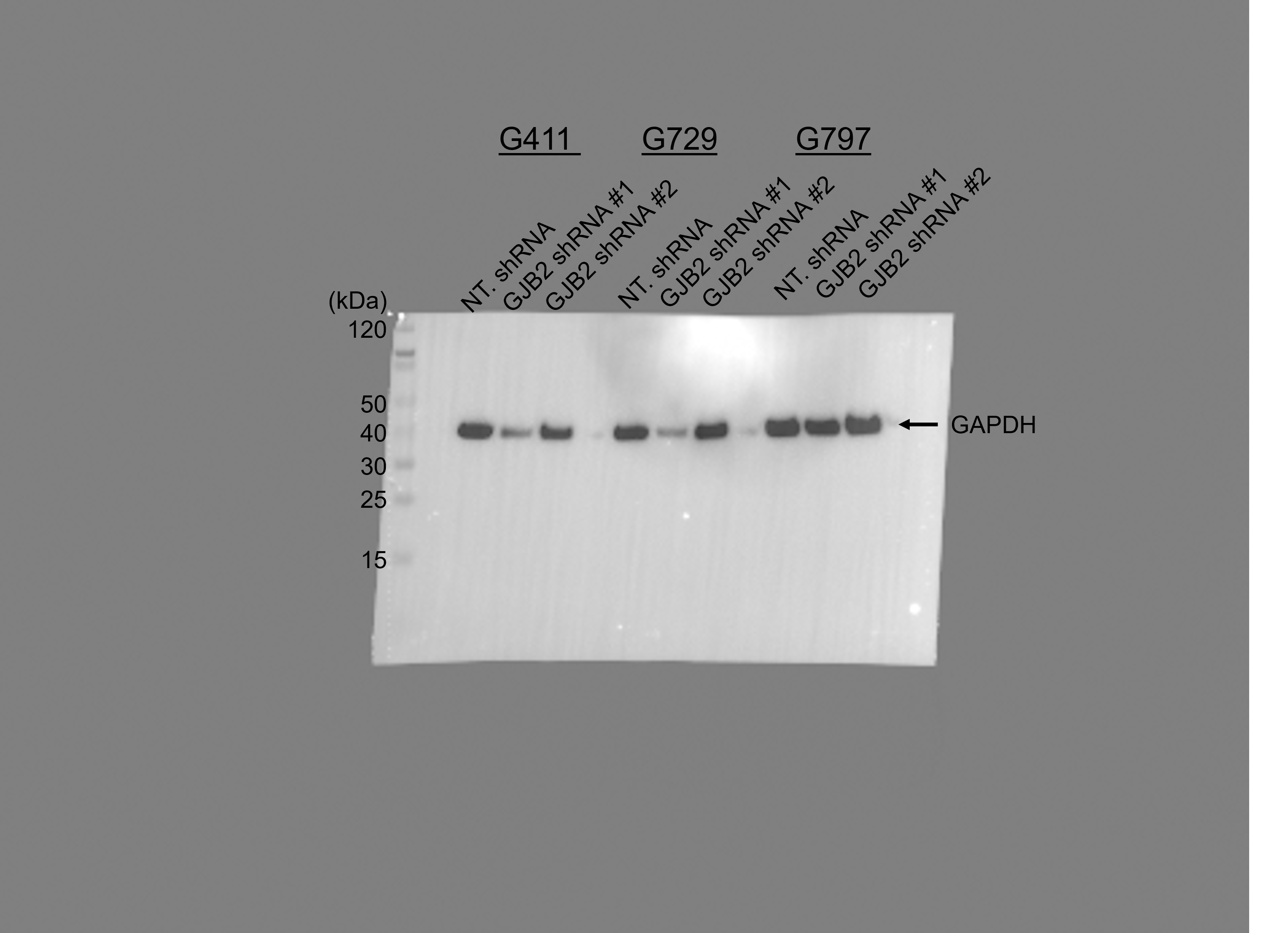

Supplement: Supplementary file 13 — Source Data Fig. 5 [file 44318_2023_16_MOESM13_ESM.zip › Figure 5/5B/G411_G729_G797_3rd replicate_GAPDH.png]

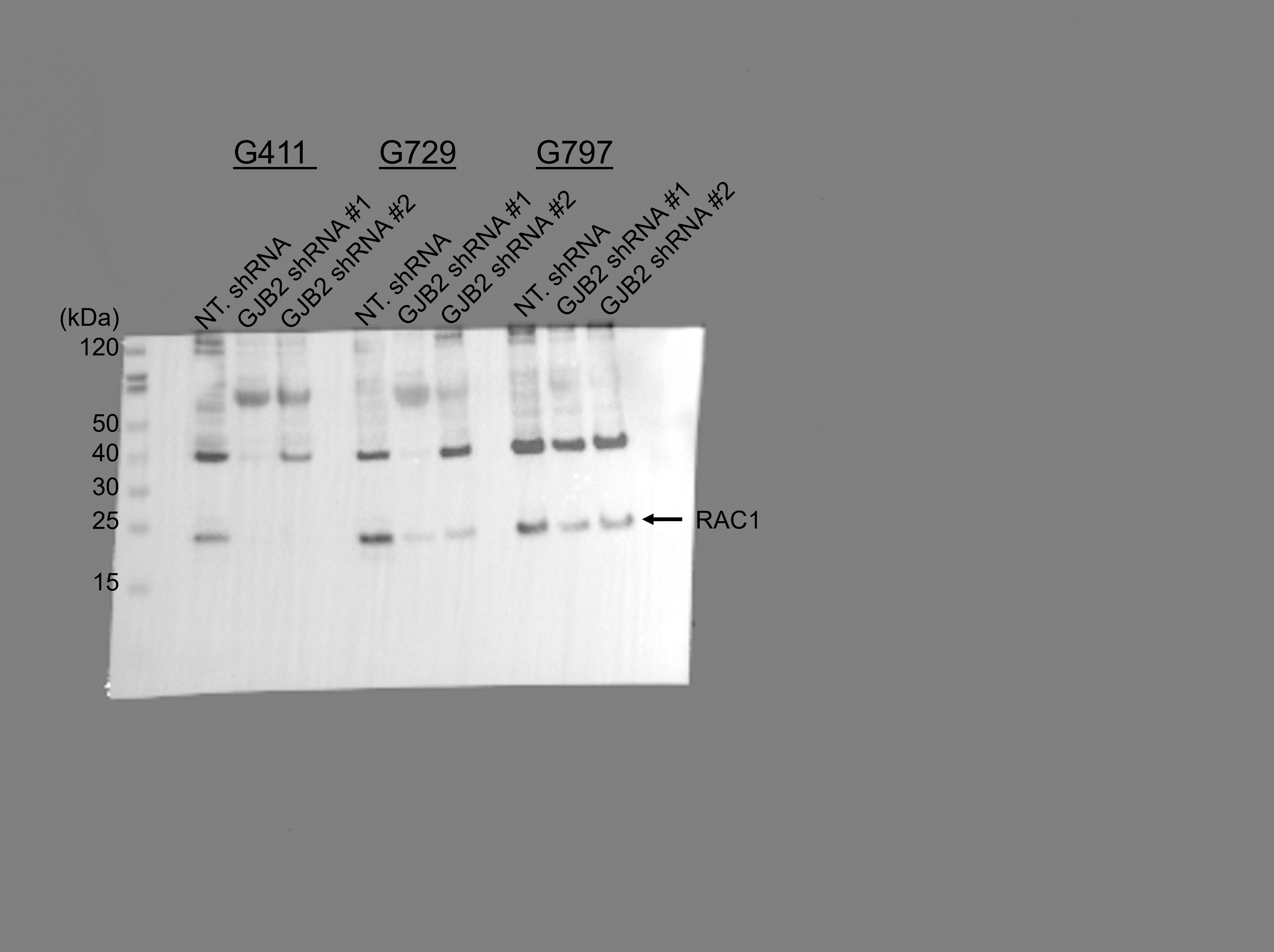

Supplement: Supplementary file 13 — Source Data Fig. 5 [file 44318_2023_16_MOESM13_ESM.zip › Figure 5/5B/G411_G729_G797_3rd replicate.png]

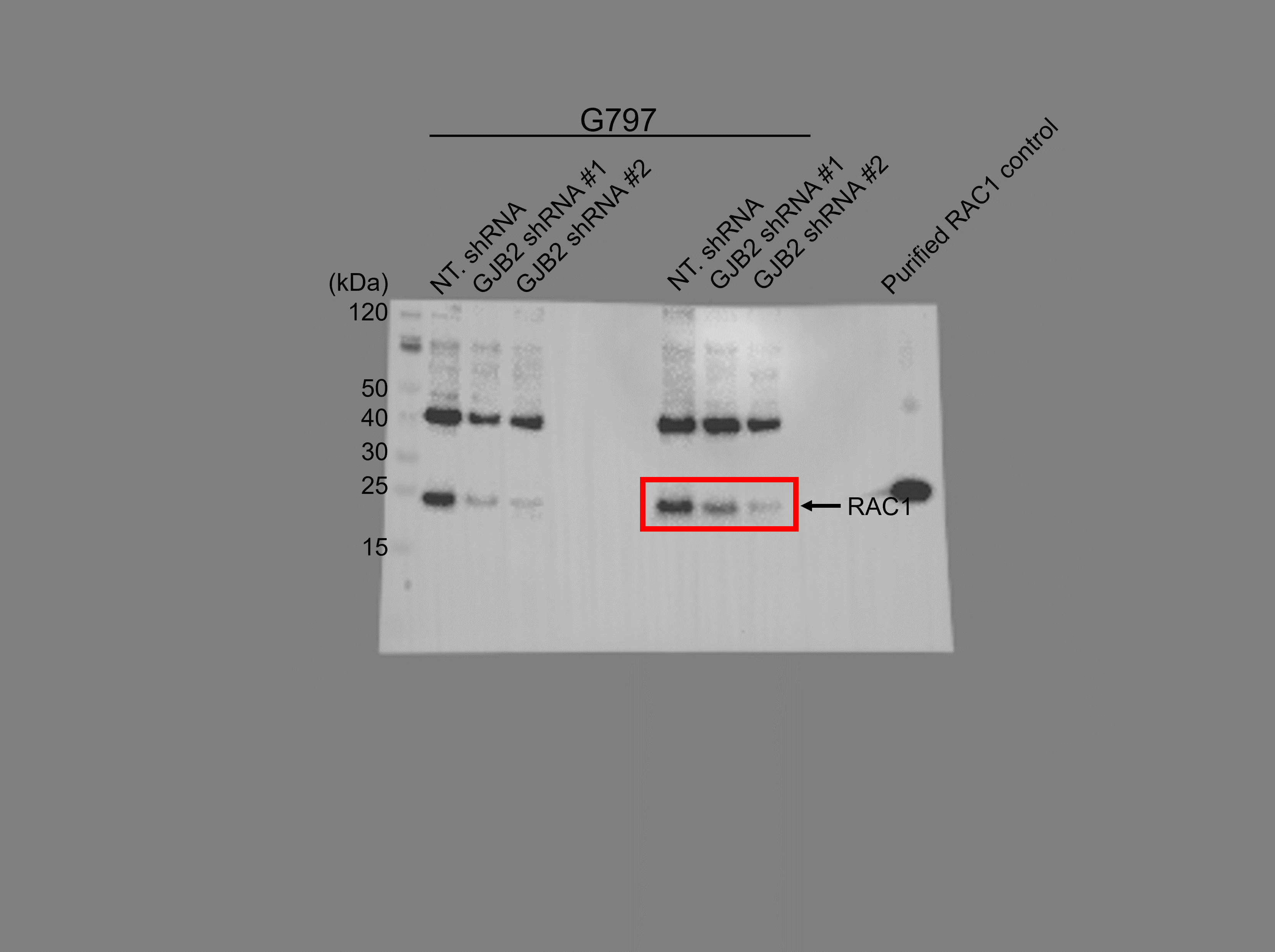

Supplement: Supplementary file 13 — Source Data Fig. 5 [file 44318_2023_16_MOESM13_ESM.zip › Figure 5/5B/G797_2 replicates.png]

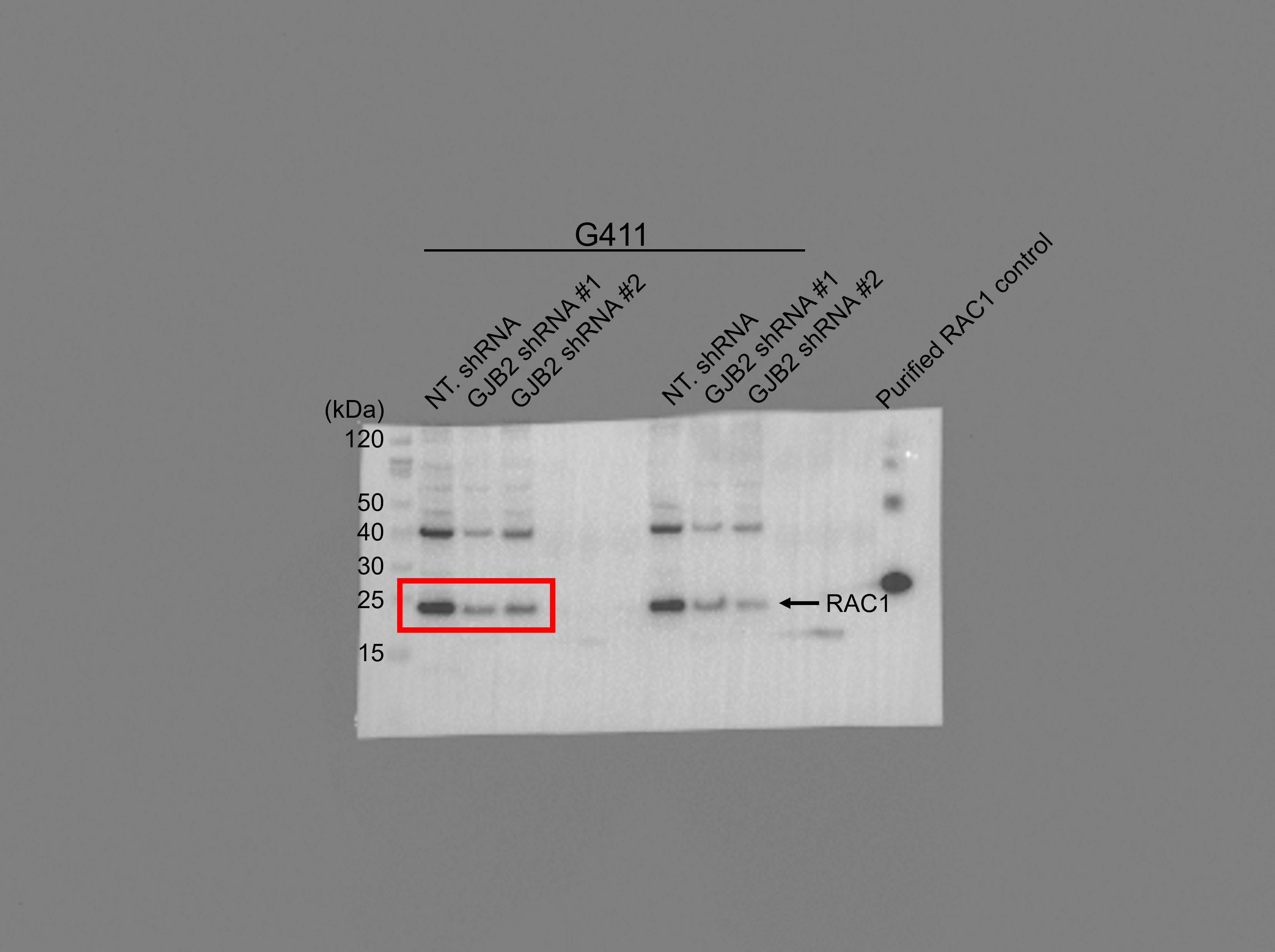

Supplement: Supplementary file 13 — Source Data Fig. 5 [file 44318_2023_16_MOESM13_ESM.zip › Figure 5/5B/G411_2 replicates.png]

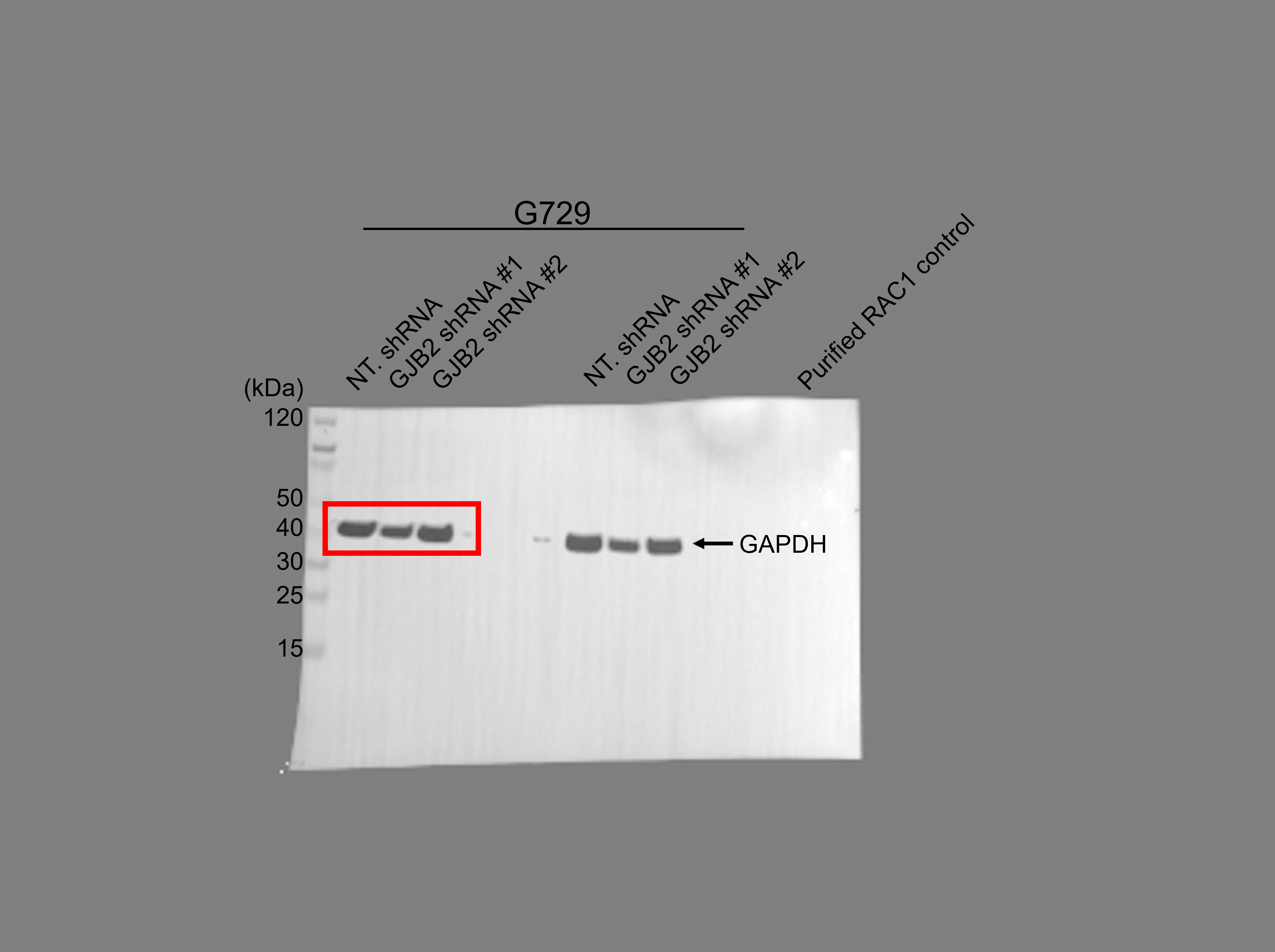

Supplement: Supplementary file 13 — Source Data Fig. 5 [file 44318_2023_16_MOESM13_ESM.zip › Figure 5/5B/G729_2 replicates_GAPDH.png]

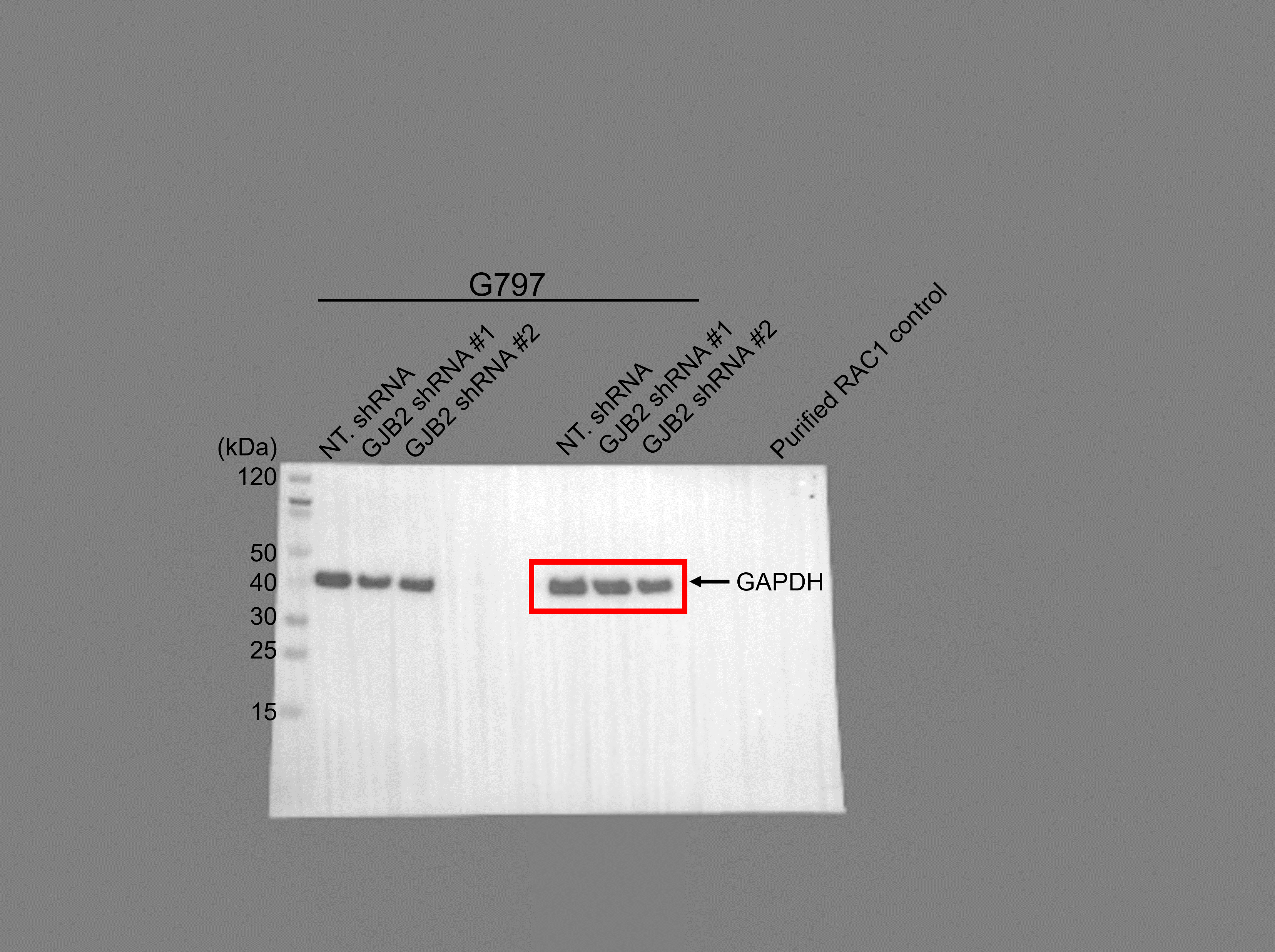

Supplement: Supplementary file 13 — Source Data Fig. 5 [file 44318_2023_16_MOESM13_ESM.zip › Figure 5/5B/G797_2 replicates_GAPDH.png]

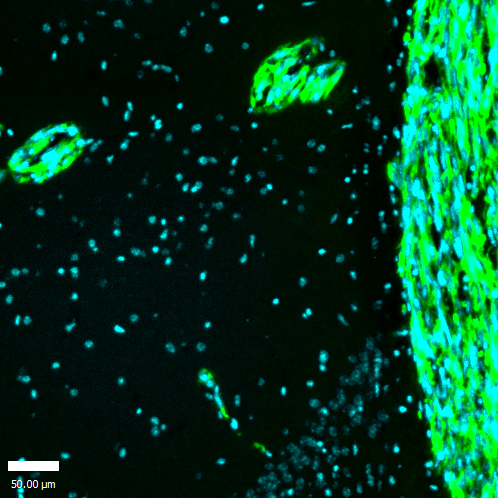

Supplement: Supplementary file 13 — Source Data Fig. 5 [file 44318_2023_16_MOESM13_ESM.zip › Figure 5/5F/Inset/GJB2 shRNA #2_Inset.tif]

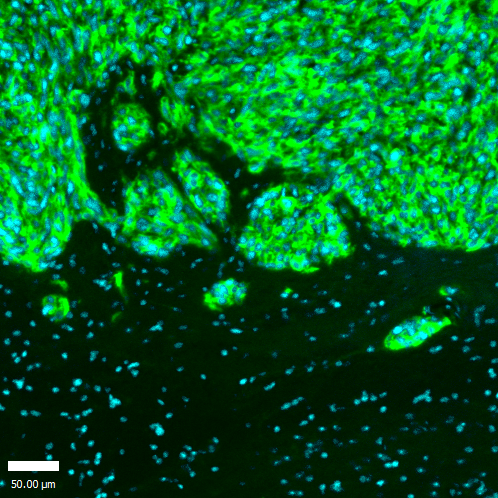

Supplement: Supplementary file 13 — Source Data Fig. 5 [file 44318_2023_16_MOESM13_ESM.zip › Figure 5/5F/Inset/NT shRNA_Inset_2.tif]

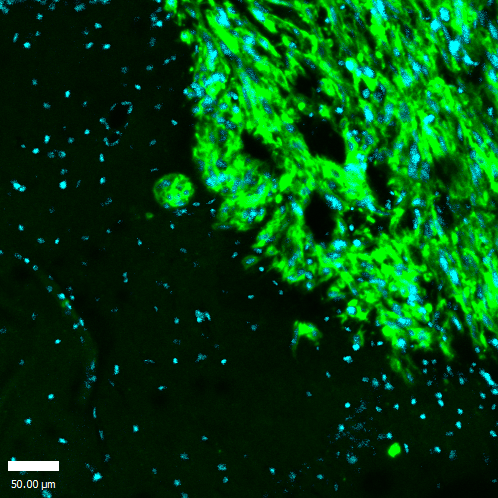

Supplement: Supplementary file 13 — Source Data Fig. 5 [file 44318_2023_16_MOESM13_ESM.zip › Figure 5/5F/Inset/GJB2 shRNA #1_Inset.tif]

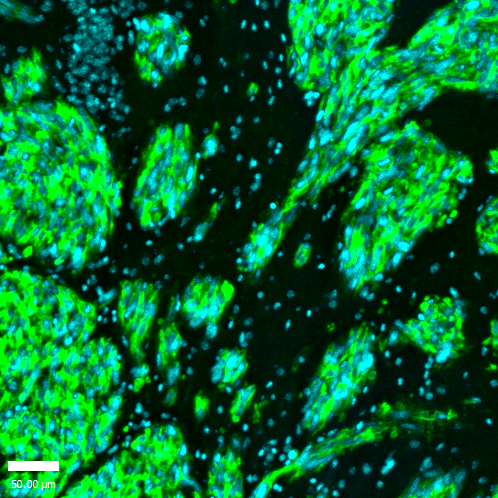

Supplement: Supplementary file 13 — Source Data Fig. 5 [file 44318_2023_16_MOESM13_ESM.zip › Figure 5/5F/Inset/NT shRNA_Inset.tif]

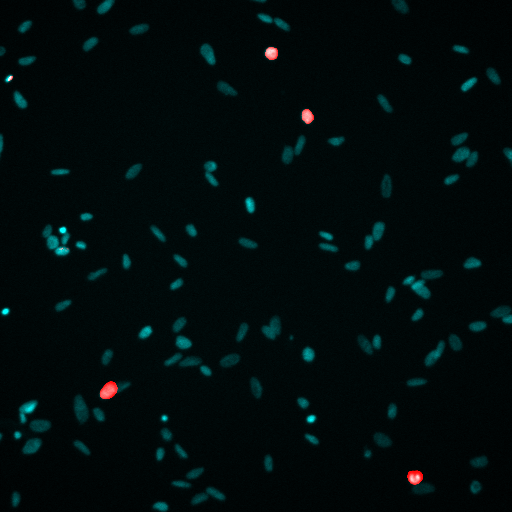

Supplement: Supplementary file 14 — Source Data Fig. 6 [file 44318_2023_16_MOESM14_ESM.zip › Figure 6/6D/G411 NT shRNA.tif]

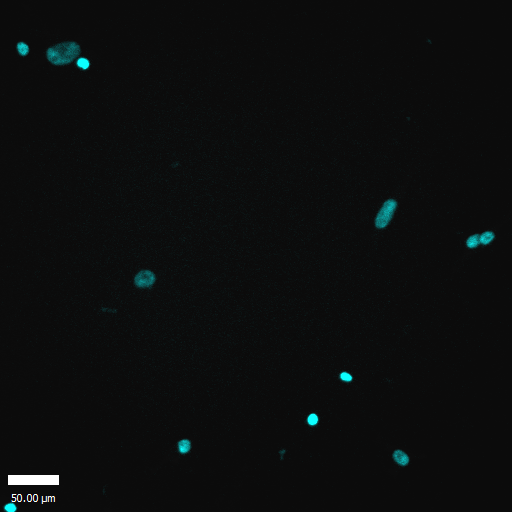

Supplement: Supplementary file 14 — Source Data Fig. 6 [file 44318_2023_16_MOESM14_ESM.zip › Figure 6/6D/G411 GJB2 shRNA #1.tif]

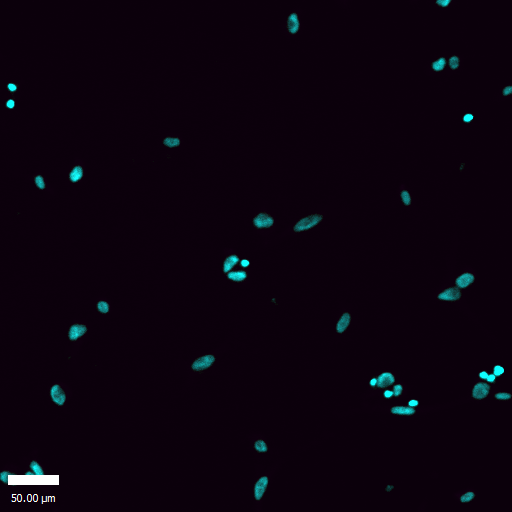

Supplement: Supplementary file 14 — Source Data Fig. 6 [file 44318_2023_16_MOESM14_ESM.zip › Figure 6/6D/G411 GJB2 shRNA #2.tif]

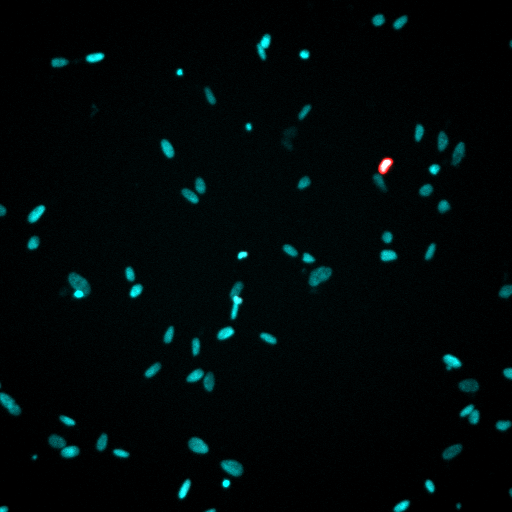

Supplement: Supplementary file 14 — Source Data Fig. 6 [file 44318_2023_16_MOESM14_ESM.zip › Figure 6/6D/G411 SCN9A shRNA #1.tif]

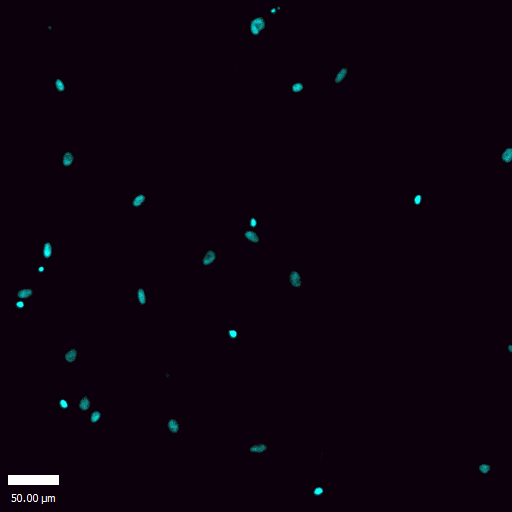

Supplement: Supplementary file 14 — Source Data Fig. 6 [file 44318_2023_16_MOESM14_ESM.zip › Figure 6/6D/G411 SCN9A shRNA #2.tif]

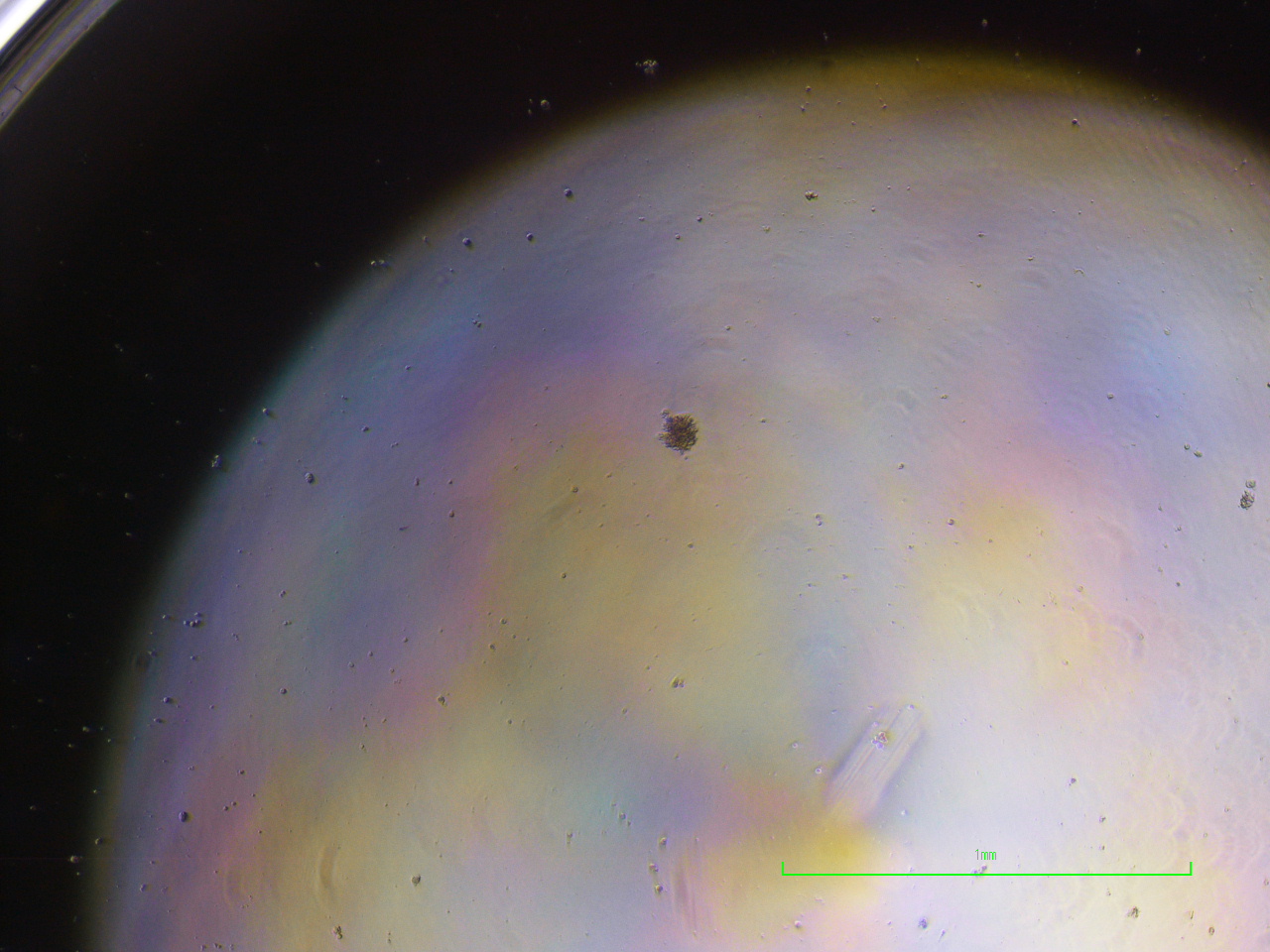

Supplement: Supplementary file 14 — Source Data Fig. 6 [file 44318_2023_16_MOESM14_ESM.zip › Figure 6/6C/G797 SCN9A shRNA #2.JPG]

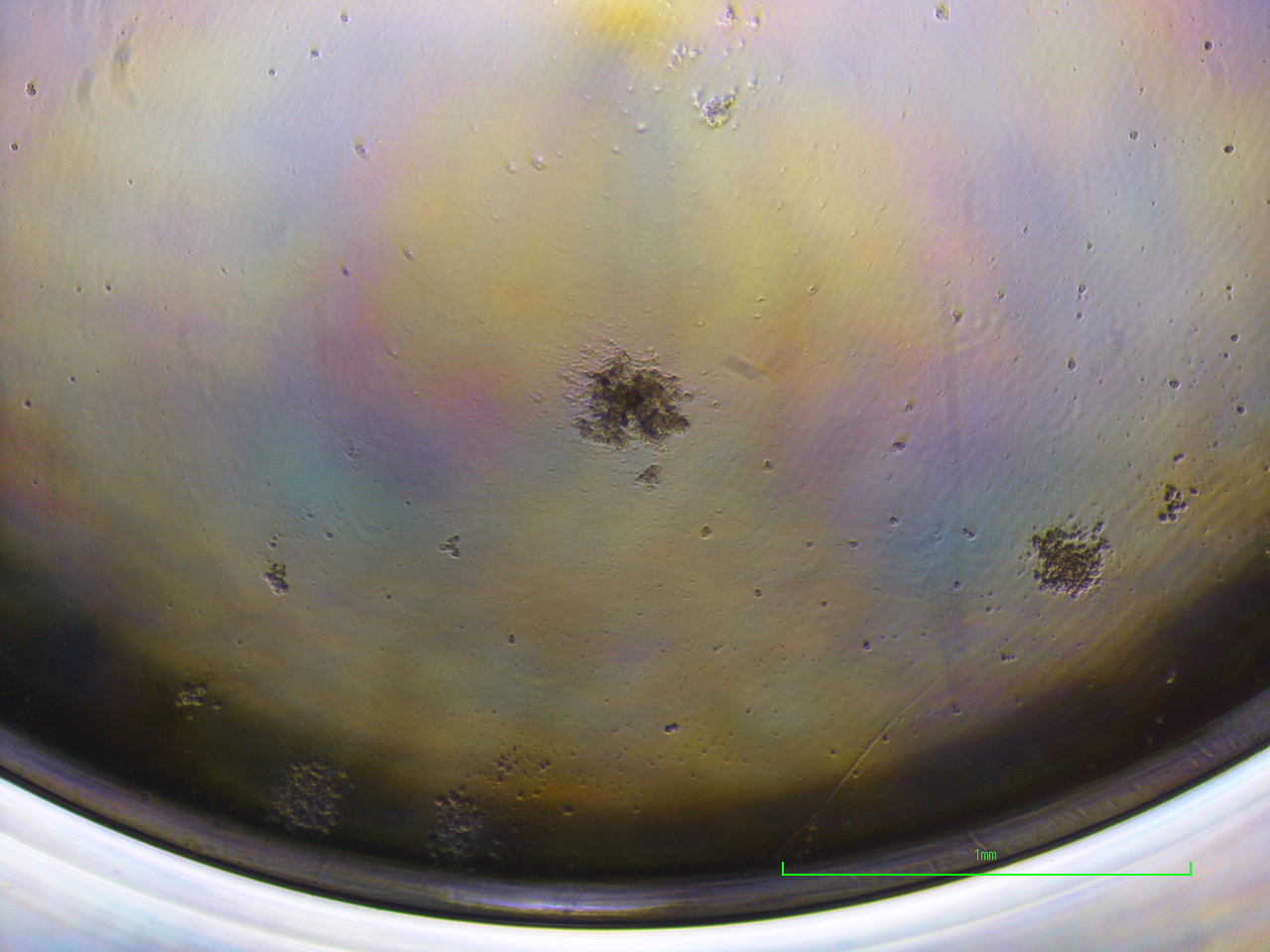

Supplement: Supplementary file 14 — Source Data Fig. 6 [file 44318_2023_16_MOESM14_ESM.zip › Figure 6/6C/G797 SCN9A shRNA #1.JPG]

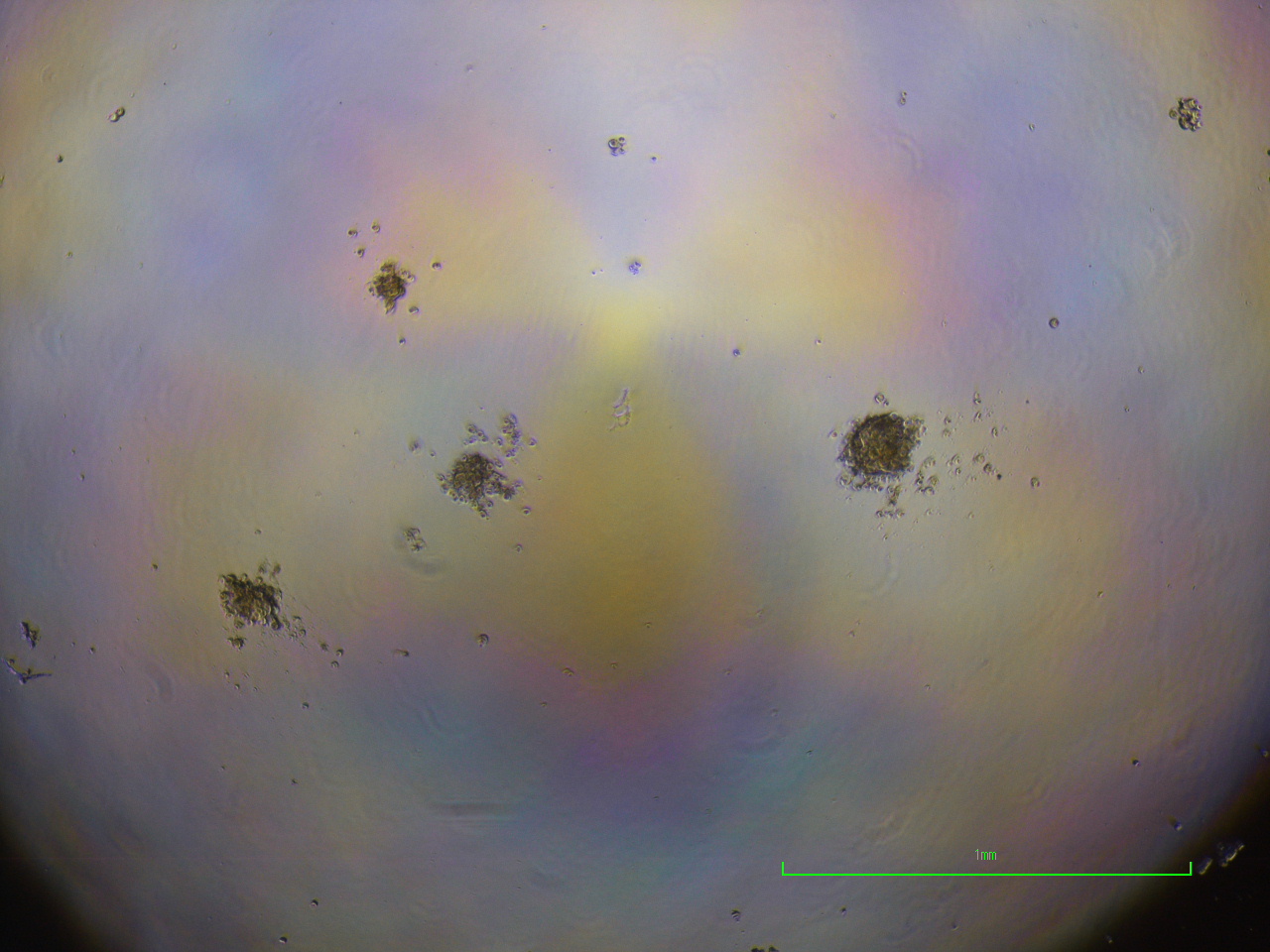

Supplement: Supplementary file 14 — Source Data Fig. 6 [file 44318_2023_16_MOESM14_ESM.zip › Figure 6/6C/G797 GJB2 shRNA #1.JPG]

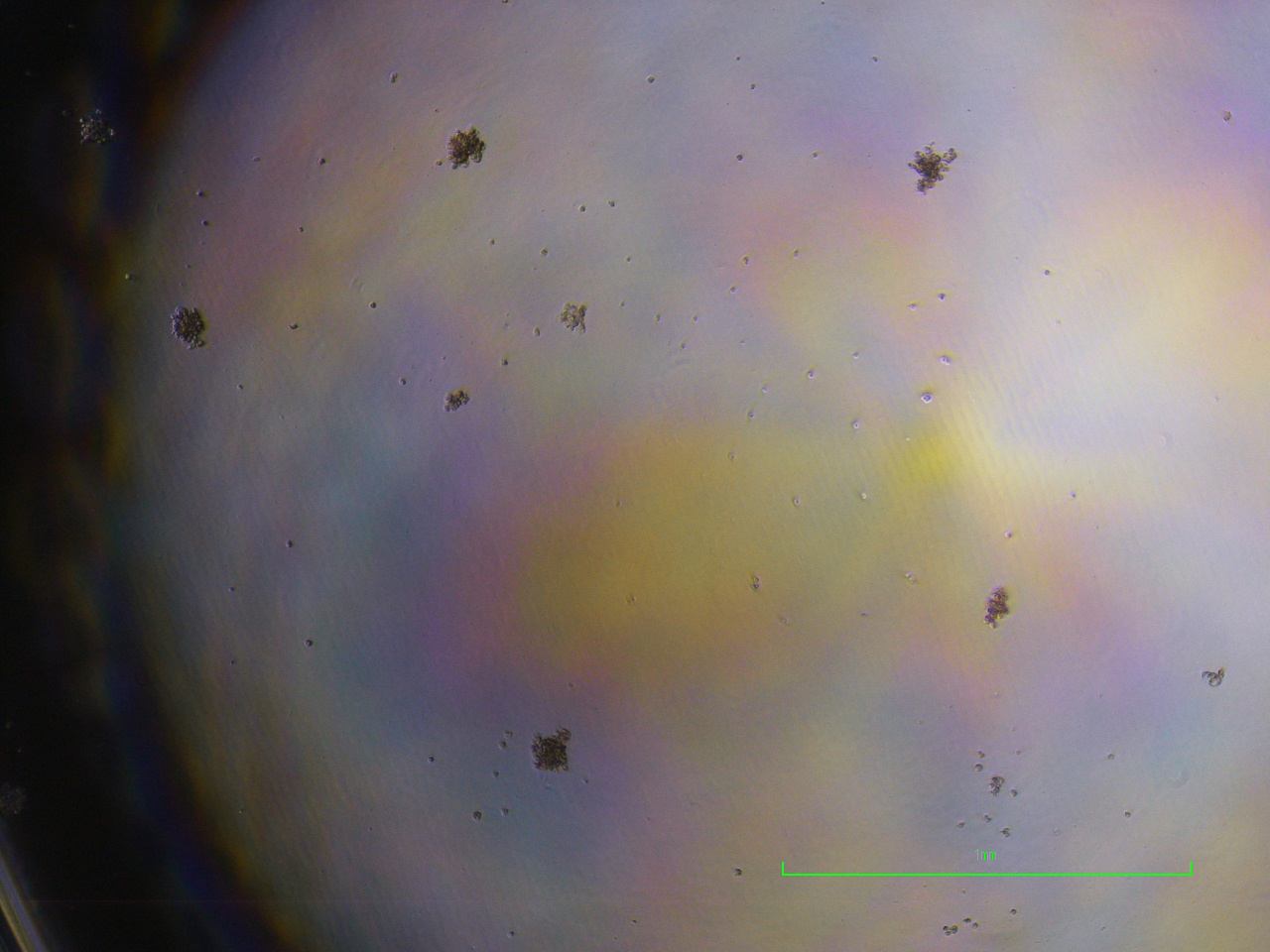

Supplement: Supplementary file 14 — Source Data Fig. 6 [file 44318_2023_16_MOESM14_ESM.zip › Figure 6/6C/G797 GJB2 shRNA #2.JPG]

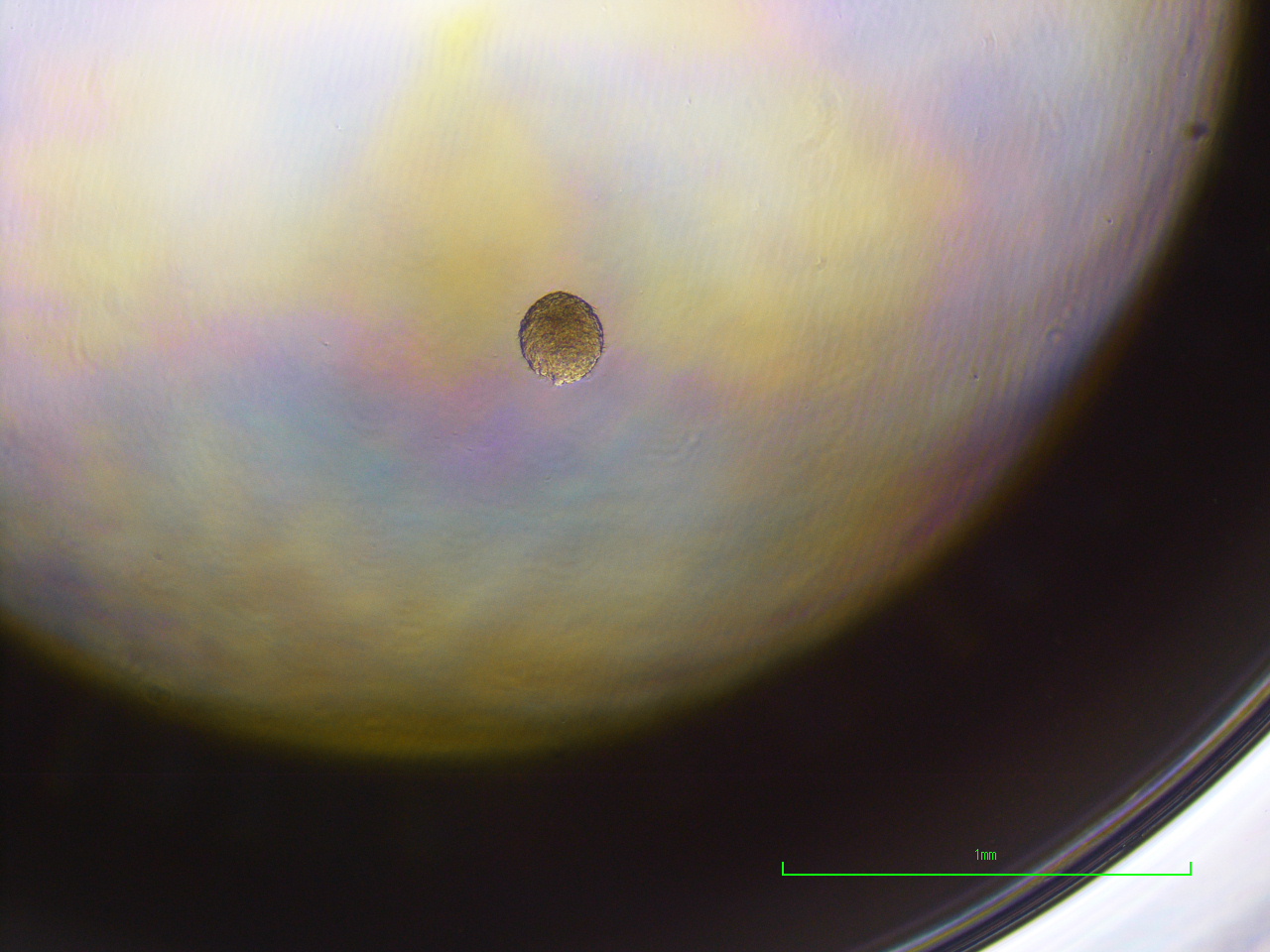

Supplement: Supplementary file 14 — Source Data Fig. 6 [file 44318_2023_16_MOESM14_ESM.zip › Figure 6/6C/G797 NT shRNA.JPG]

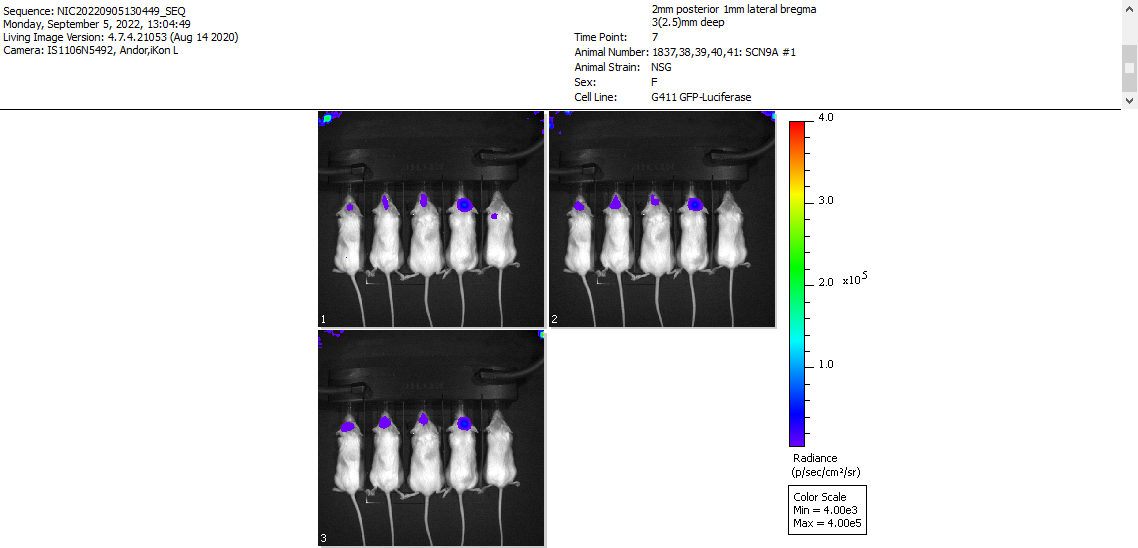

Supplement: Supplementary file 14 — Source Data Fig. 6 [file 44318_2023_16_MOESM14_ESM.zip › Figure 6/6E/SCN9A/Day 7/S1 1837-41.png]

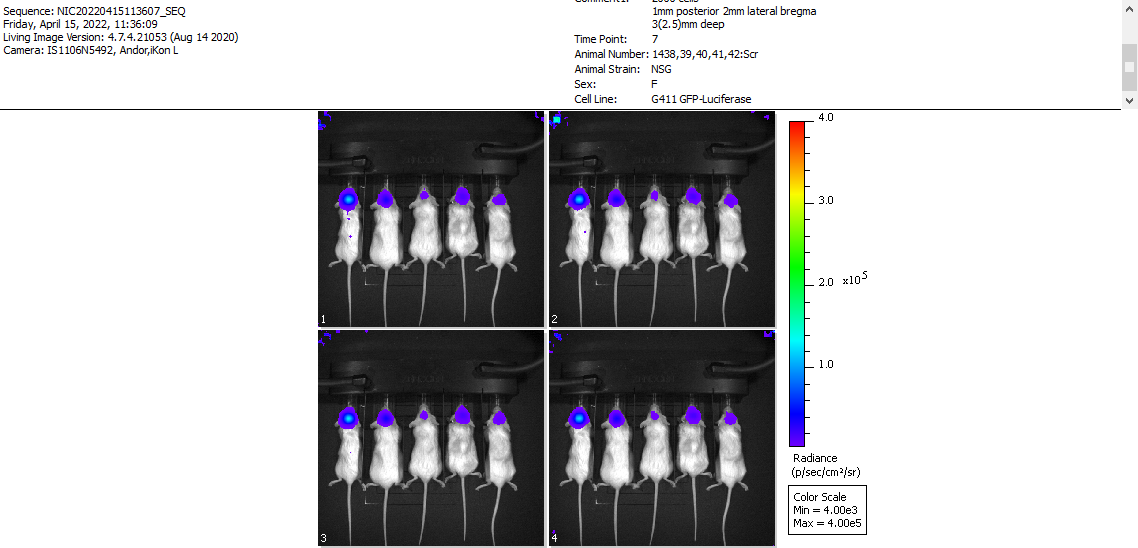

Supplement: Supplementary file 14 — Source Data Fig. 6 [file 44318_2023_16_MOESM14_ESM.zip › Figure 6/6E/SCN9A/Day 7/NT 1438-42.png]

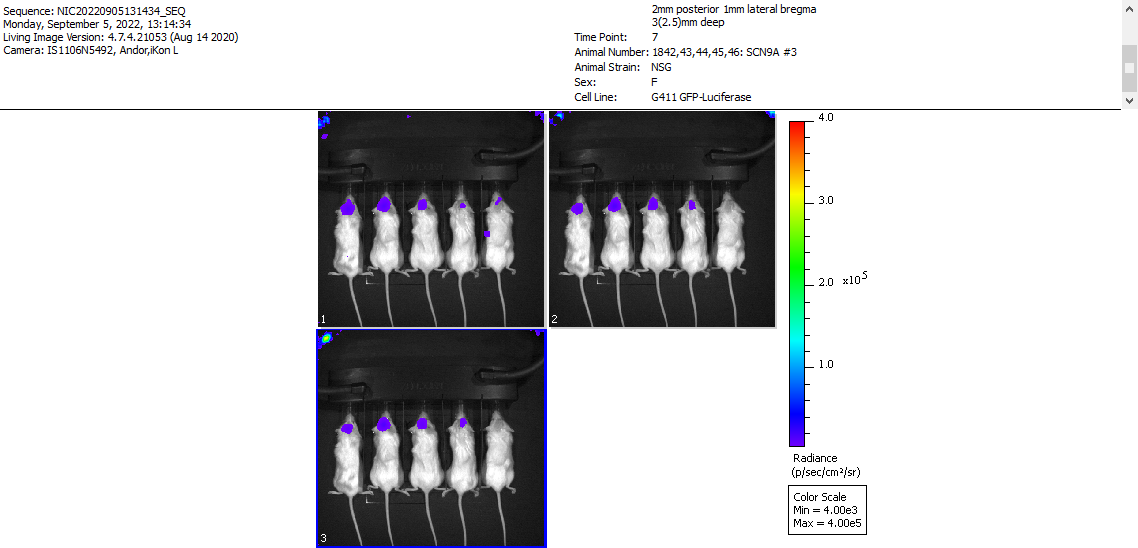

Supplement: Supplementary file 14 — Source Data Fig. 6 [file 44318_2023_16_MOESM14_ESM.zip › Figure 6/6E/SCN9A/Day 7/S3 1842-46.png]

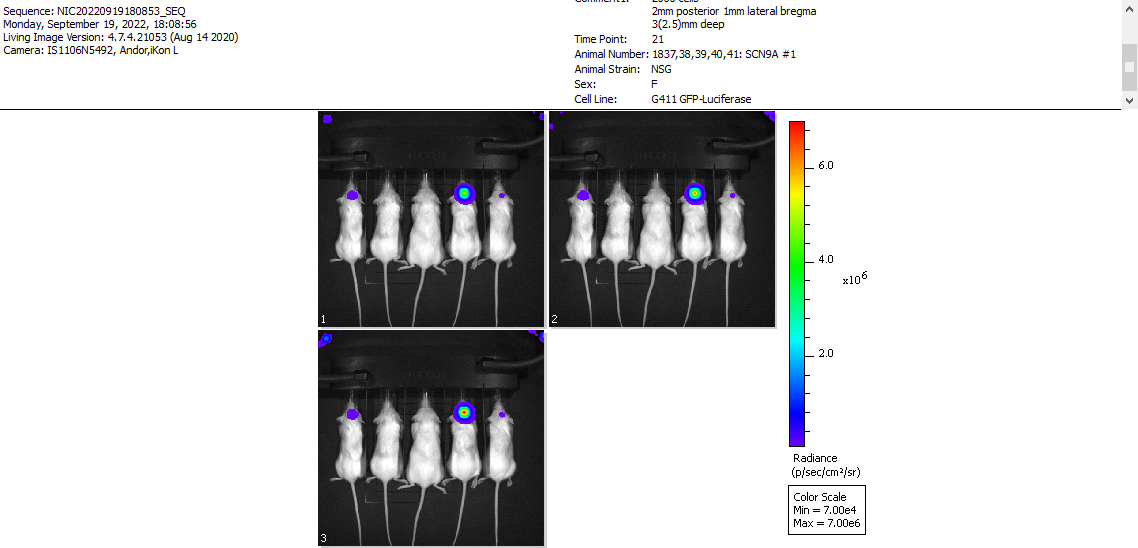

Supplement: Supplementary file 14 — Source Data Fig. 6 [file 44318_2023_16_MOESM14_ESM.zip › Figure 6/6E/SCN9A/Day 21/S1 1837-41.png]

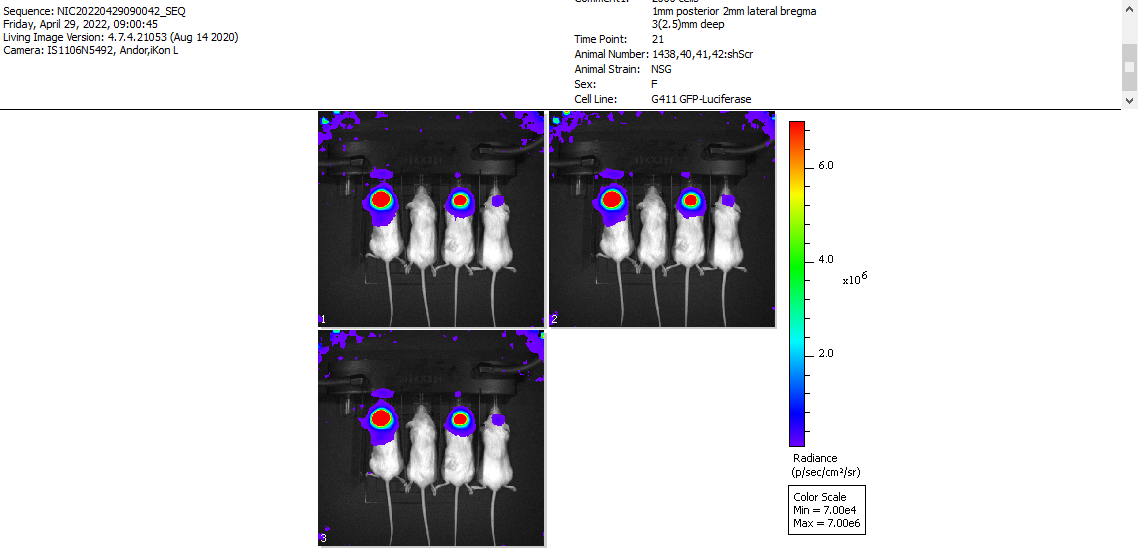

Supplement: Supplementary file 14 — Source Data Fig. 6 [file 44318_2023_16_MOESM14_ESM.zip › Figure 6/6E/SCN9A/Day 21/NT 1438-42.png]

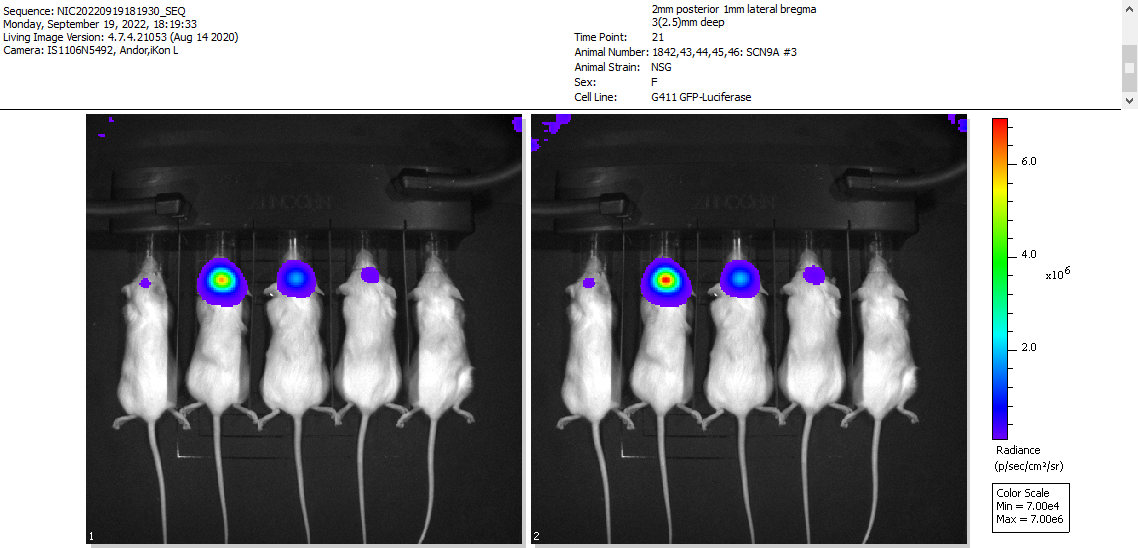

Supplement: Supplementary file 14 — Source Data Fig. 6 [file 44318_2023_16_MOESM14_ESM.zip › Figure 6/6E/SCN9A/Day 21/S3 1842-46.png]

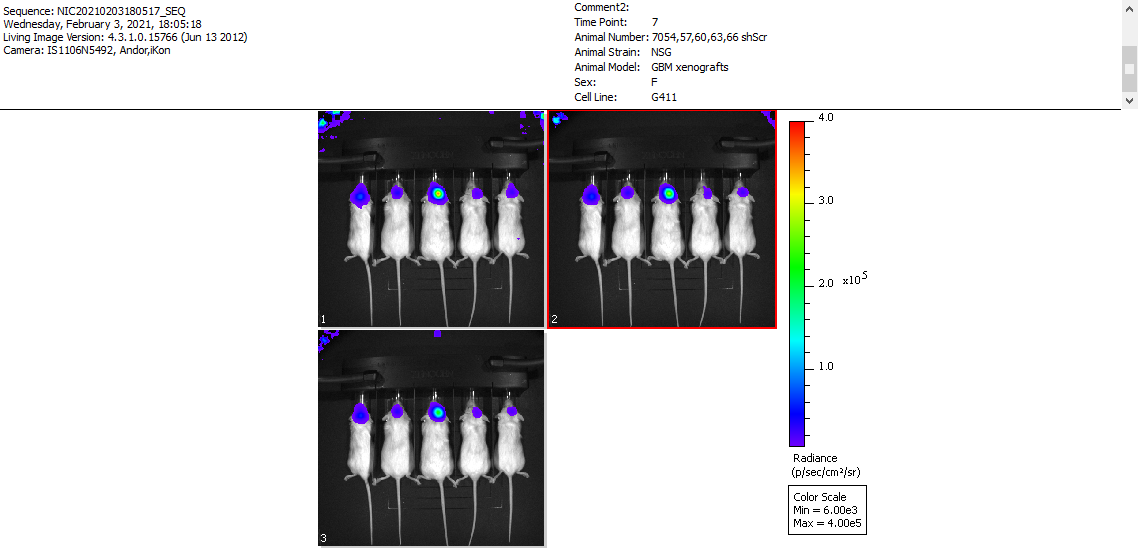

Supplement: Supplementary file 14 — Source Data Fig. 6 [file 44318_2023_16_MOESM14_ESM.zip › Figure 6/6E/GJB2/Day 7/NT shRNA 7054-66.png]

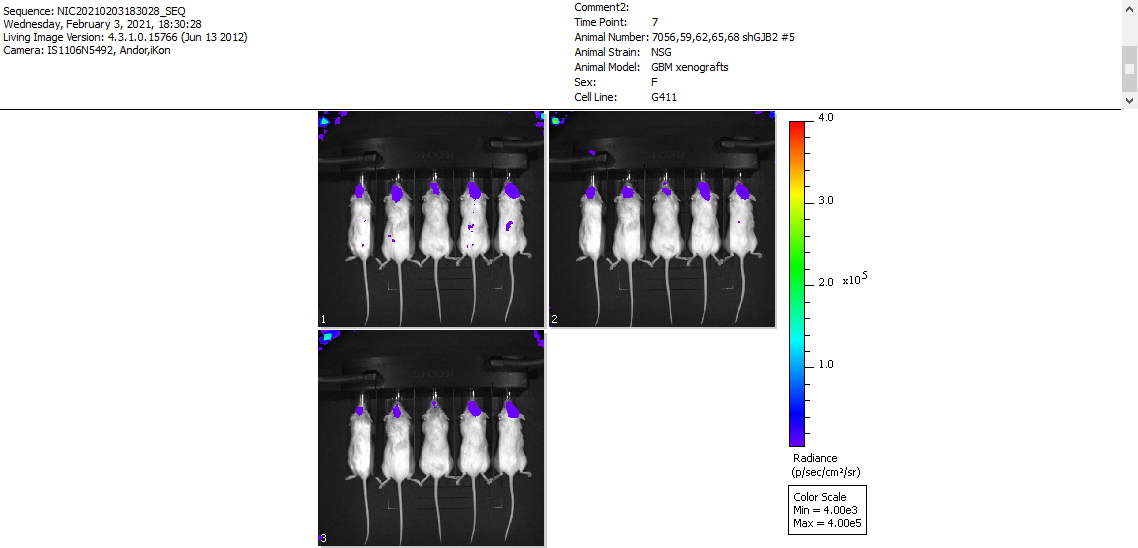

Supplement: Supplementary file 14 — Source Data Fig. 6 [file 44318_2023_16_MOESM14_ESM.zip › Figure 6/6E/GJB2/Day 7/GJB2 shRNA #2 7056-68.png]

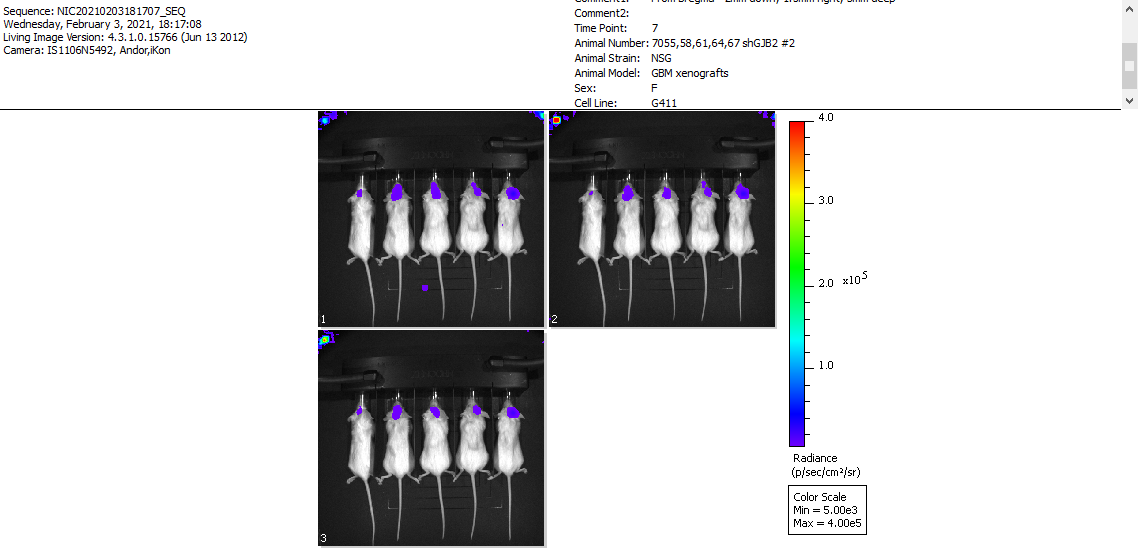

Supplement: Supplementary file 14 — Source Data Fig. 6 [file 44318_2023_16_MOESM14_ESM.zip › Figure 6/6E/GJB2/Day 7/GJB2 shRNA #1 7055-67.png]

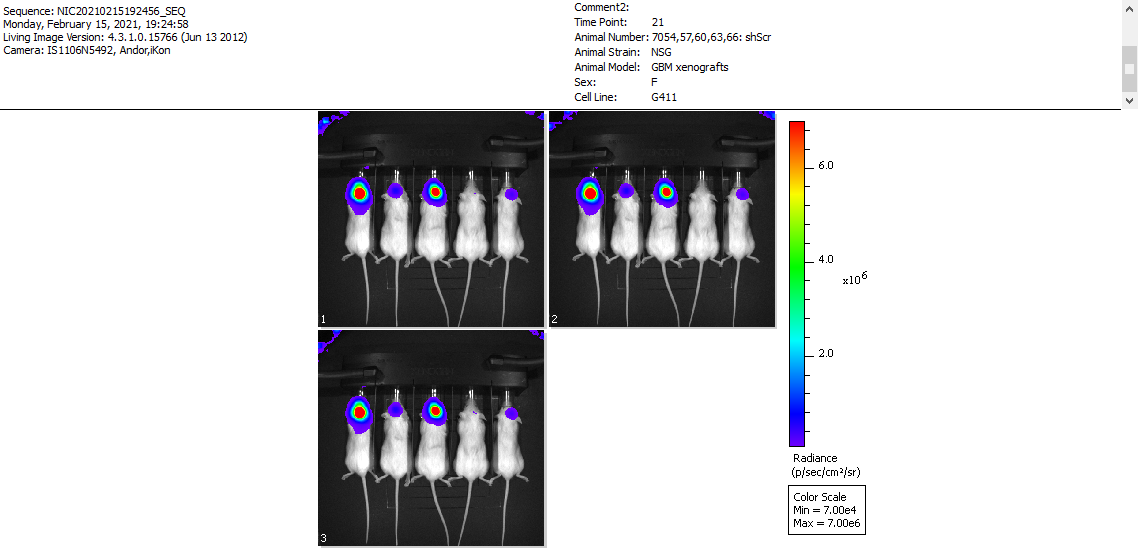

Supplement: Supplementary file 14 — Source Data Fig. 6 [file 44318_2023_16_MOESM14_ESM.zip › Figure 6/6E/GJB2/Day 21/NT shRNA 7054-66.png]

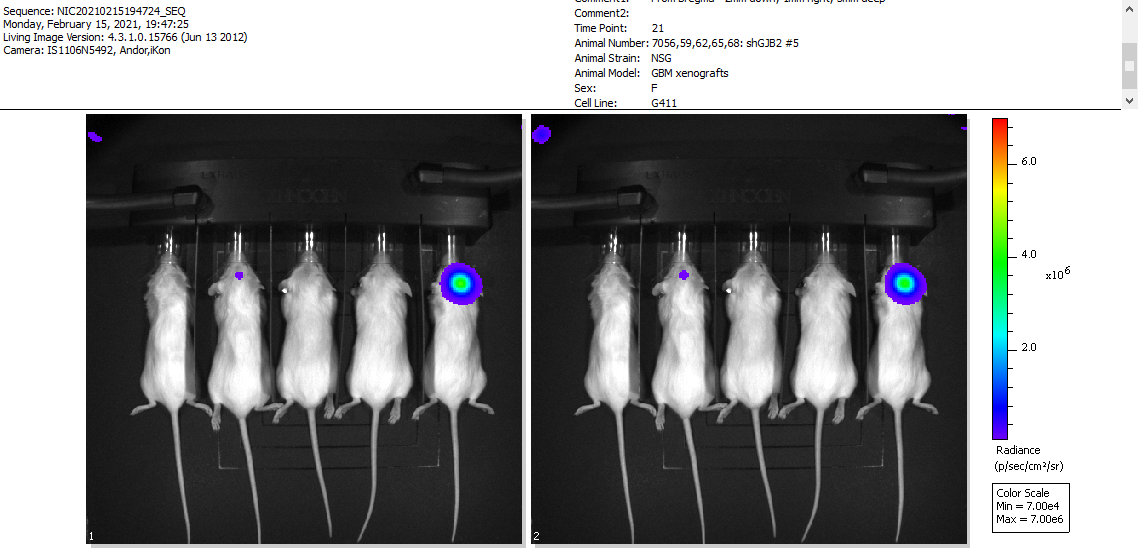

Supplement: Supplementary file 14 — Source Data Fig. 6 [file 44318_2023_16_MOESM14_ESM.zip › Figure 6/6E/GJB2/Day 21/GJB2 shRNA #2 7056-68.png]

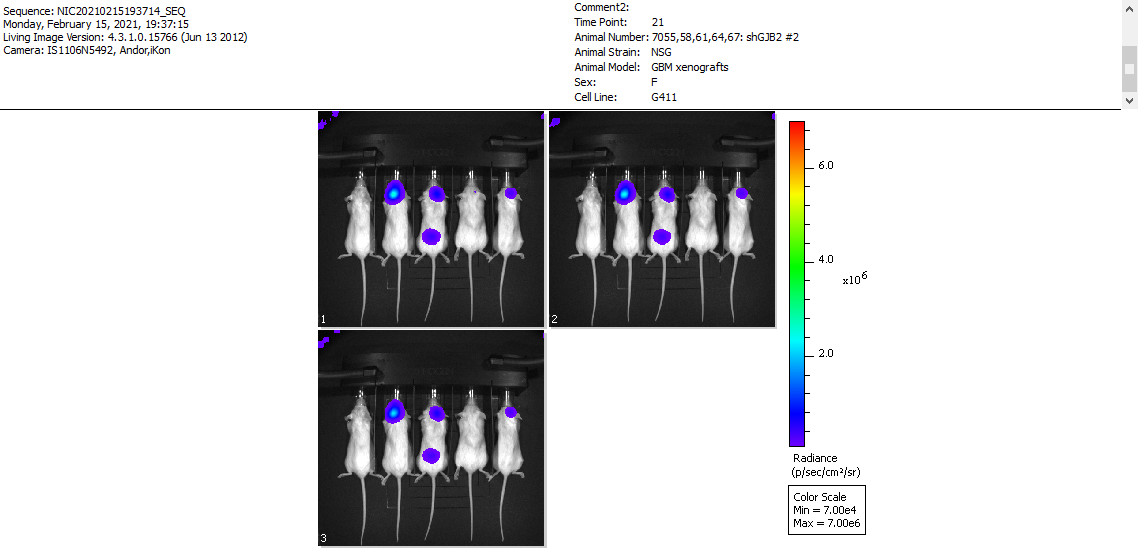

Supplement: Supplementary file 14 — Source Data Fig. 6 [file 44318_2023_16_MOESM14_ESM.zip › Figure 6/6E/GJB2/Day 21/GJB2 shRNA #1 7055-67.png]
