# Supplementary material for: Pan-cancer ion transport signature reveals functional regulators of glioblastoma aggression
Source: EMBO J. 2024 Jan 2;43(2):196–224. doi: 10.1038/s44318-023-00016-x (PMC10897389; doi:10.1038/s44318-023-00016-x)
Supplement: Supplementary file 17 — Expanded View Figures [file 44318_2023_16_MOESM17_ESM.pdf]

Expanded View Figures

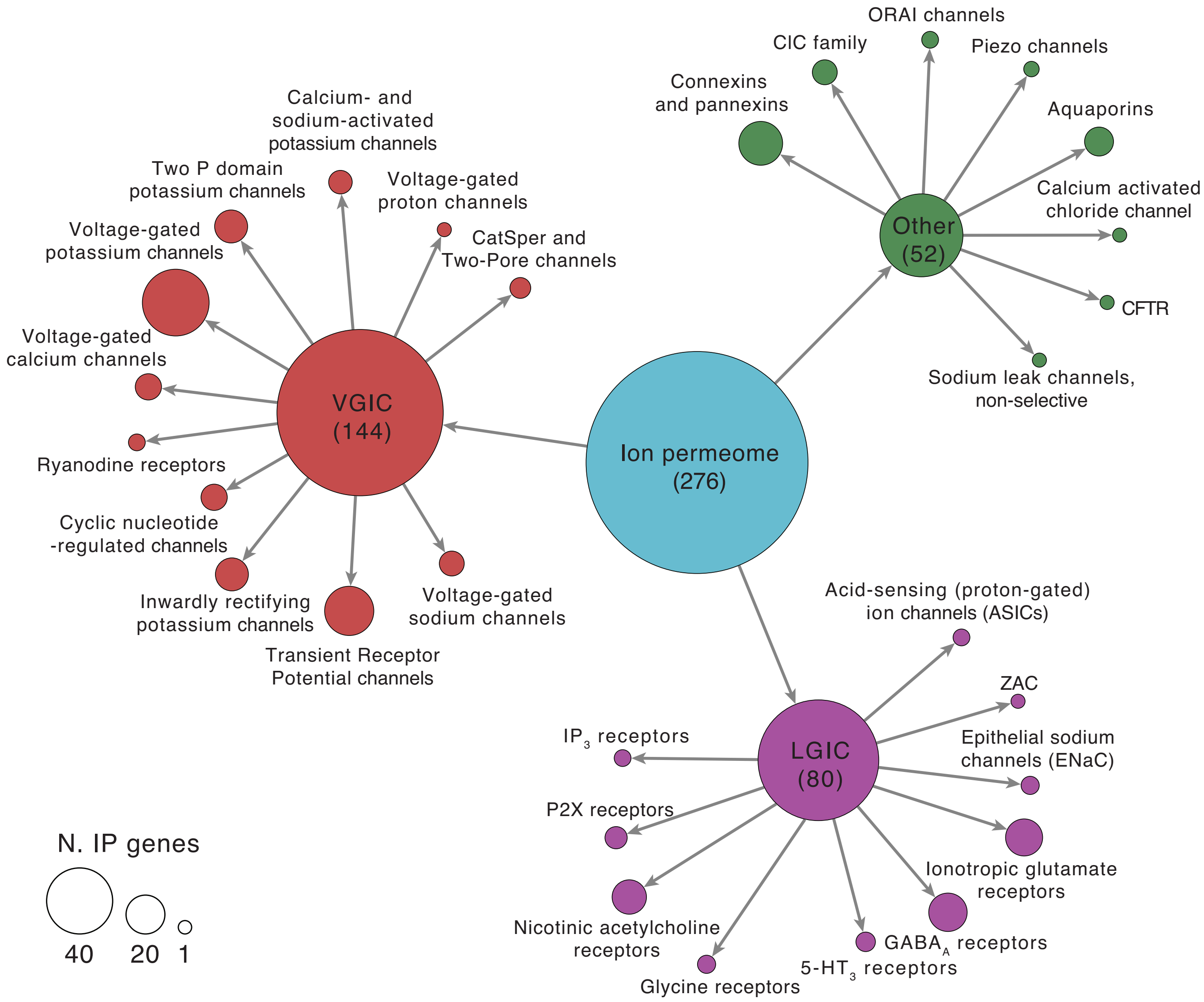

Figure EV1. Classification of ion permeome (IP) genes used in our study.

Genes encoding IP proteins with known specific inhibitors were obtained from the Guide to Pharmacology database and filtered to include genes with expression profiles in TCGA. The network shows a hierarchical classification of IP proteins by type or family (nodes) with arrows indicating subclasses derived from larger classes. Node size reflects the number of genes within each type or family. Node color reflects major IP gene classes. Other ion channels (IC), voltage-gated ICs (VGIC), and ligand-gated ICs (LGICs) represent the major classes shown. Source data are available online for this figure.

 © The Author(s)

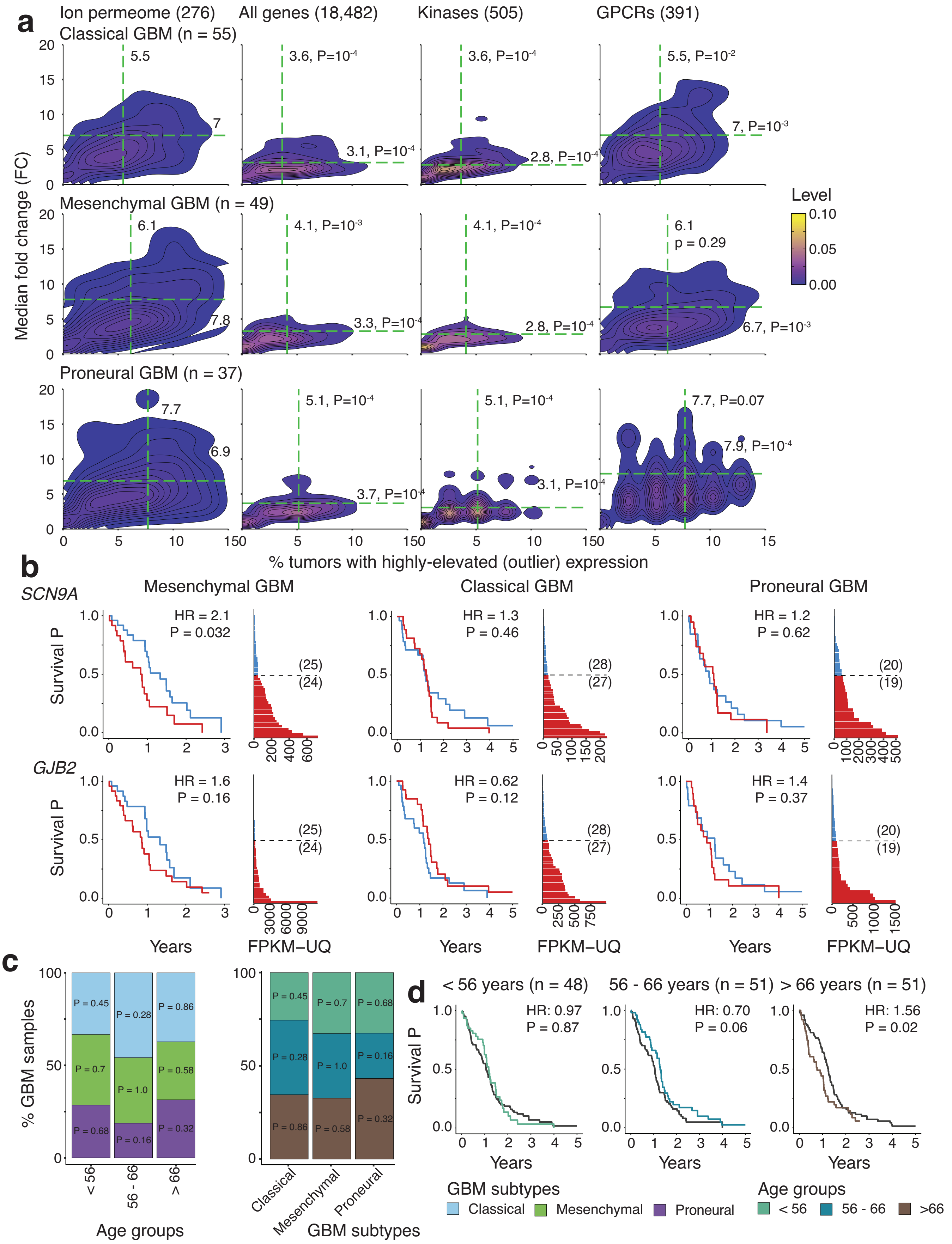
a
Ion permeome (276)
All genes (18,482)
Kinases (505)
GPCRs (391)
Classical GBM (n = 55)
Mesenchymal GBM (n = 49)
Proneural GBM (n = 37)
Median fold change (FC)
Level
0.10
0.05
0.00
% tumors with highly-elevated (outlier) expression
b
SCN9A
GJB2
Mesenchymal GBM
Classical GBM
Proneural GBM
Survival P
Years
FPKM–UQ
c
% GBM samples
Age groups
GBM subtypes
Classical
Mesenchymal
Proneural
d
< 56 years (n = 48)
56 - 66 years (n = 51)
> 66 years (n = 51)
Survival P
Years
Age groups
< 56
56 - 66
>66

Figure EV2. GBM subtype analysis of IP gene expression and patient survival.

(**A**) High upregulation of IP genes in classical, mesenchymal, and proneural subtypes of GBM. Density plots show the joint distribution of gene expression increase (fold-change (FC), log2) and the fraction of affected cancer samples. IP genes (left) are compared to three controls (middle to right): (i) all protein-coding genes, and two classes of drug targets: (ii) kinases and (iii) GPCRs, with down-sampling performed similarly to the main analysis. Dashed green lines show median values. Representative iterations with median fold-change are shown. (**B**) Kaplan-Meier plots of overall survival (OS) in mesenchymal (left), classical (middle), and proneural (right) GBMs grouped by *GJB2* or *SCN9A* expression in TCGA. Bar plots show gene expression levels in risk groups. Risk groups were determined by median dichotomisation for *GJB2* and *SCN9A* expression levels. Wald *P*-values, Univariate HR values, and sample counts are shown. Patient age was included as a covariate in TCGA analyses. (**C**) GBM subtype associations with patient age. Stacked bar plots show the fraction of samples from GBM subtypes (left) or associated ages of patients (right) in each age group or GBM subtype. Mann-Whitney U-test *P*-values are shown. (**D**) Kaplan-Meier plots of OS in all TCGA GBM patients grouped by patient age. Patient groups were determined as three equally-sized groups based on low, medium, or high age. Wald *P*-values, Univariate HR values, and sample counts are shown. Source data are available online for this figure

 © The Author(s)

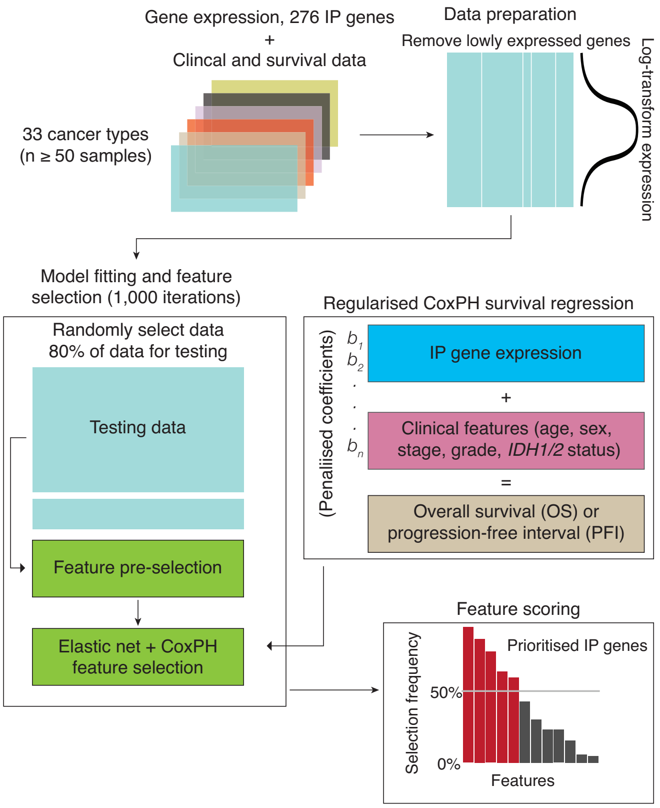

Figure EV3. Overview of the machine learning pipeline for identifying survival-associated IP genes.

IP gene expression was evaluated for significant survival associations individually for 33 cancer types from TCGA. IP gene expression values were log-normalized and used as features in a machine learning framework. Regularized CoxPH models were trained on iterations of 80% of samples within each cancer type, using IP gene expression values and clinical variables (patient age and sex, tumor stage, grade, and *IDH1/2* mutation status) as model features and patient survival as the response variables. Within each iteration, features were first pre-selected using univariate CoxPH models trained, and only genes significantly associated with patient survival ($P < 0.1$, Wald test) were selected for the multivariate model. After 1,000 iterations of feature prioritisation, IP genes associated with patient survival in at least 50% of models were selected as high-confidence hits. Source data are available online for this figure

© The Author(s)

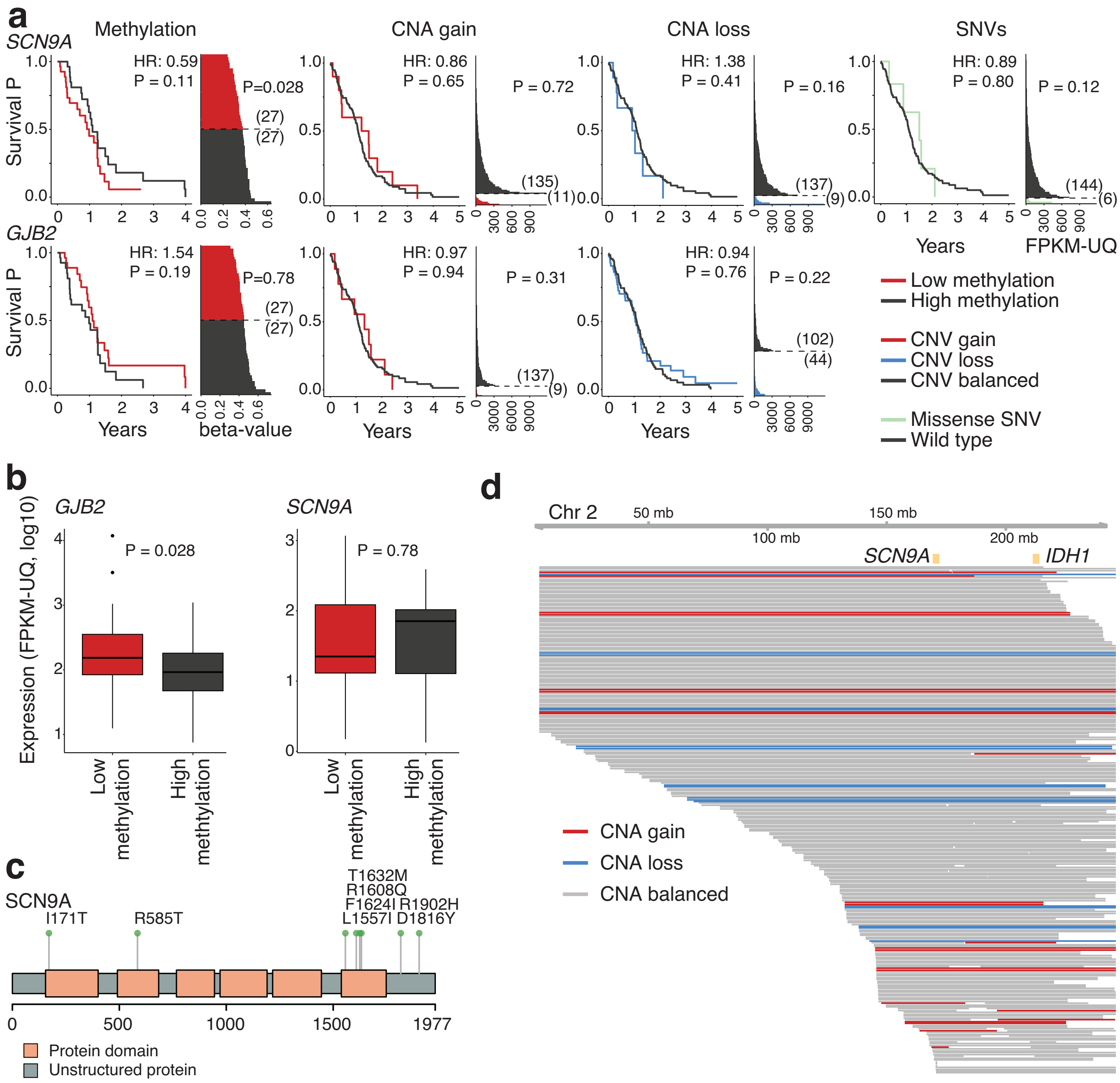

Figure EV4. Genetic alterations and DNA methylation of *GJB2* and *SCN9A* have few associations with gene expression.

(**A**) Kaplan-Meier plots of OS in GBM patients (left to right): *GJB2* and *SCN9A* promoter DNA methylation; relative somatic copy number alterations (CNAs), and somatic single nucleotide variants (SNVs) in GBMs in TCGA. Bar plots show mean gene promoter methylation (left, beta-values) or gene expression in risk groups. Risk groups were determined by median dichotomisation for promoter methylation and presence or absence of a CNAs or SNVs. Wald P-values, Univariate HR values, and sample counts are shown. (**B**) IP gene expression associations with promoter methylation. Boxplots show gene *GJB2* and *SCN9A* gene expression in samples with high and low gene promoter methylation. Methylation groups were defined by median dichotomisation of mean methylation beta values of each promoter. Box plots span the interquartile range (IQR; 25th-75th percentiles) where median values are shown as lines and whiskers reflect values within 1.5x of IQR. (**C**) Non-silent SNVs in *SCN9A* in TCGA GBMs. Patients from panel (a) with missense SNVs in *SCN9A* are shown with the reference and alternate amino acids. (**D**) CNAs in *SCN9A* in GBM associate with CNAs in the adjacent *IDH1*. CNAs for GBM TCGA samples are included with labels showing the genomic loci of *SCN9A* and *IDH1*. Source data are available online for this figure

 © The Author(s)

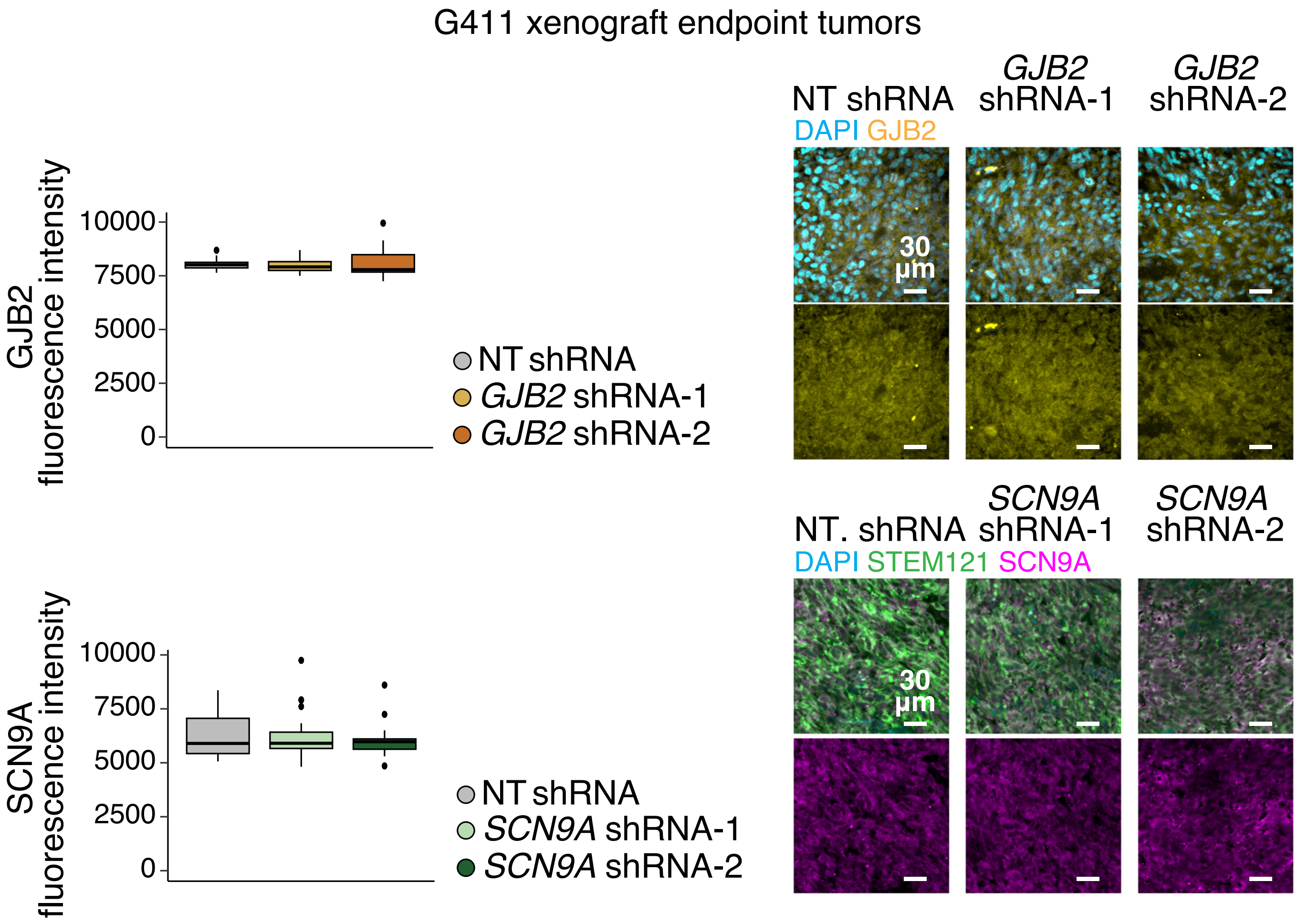

Figure EV5. Endpoint GBM tumors in the knockdown groups are shRNA escapers.

Tumors with knockdown of *GJB2* or *SCN9A* at endpoint have comparable expression of GJB2 or SCN9A. Brains from or IP gene knockdown xenograft mice or NT control xenograft mice were harvested at humane endpoint from tumor outgrowth. Immunofluorescence imaging of GJB2 or SCN9A were performed on *GJB2* or *SCN9A* knockdown tumors, respectively. For GJB2 quantification, DAPI density was used to identify tumor regions because the GJB2 and STEM121 antibodies were both from mouse. For SCN9A quantification, tumor regions were identified by co-staining with human-specific antibody STEM121. Results are from $n = 4$ mice for NT control group, $n = 3$ mice for each *GJB2* knockdown group, and $n = 4$ mice for each *SCN9A* knockdown group. All groups were not significantly different according to Welch's T-tests. Box plots span the interquartile range (IQR; 25th-75th percentiles) where median values are shown as lines and whiskers reflect values within 1.5x of IQR. Source data are available online for this figure

© The Author(s)
